# The RNA Demethylases ALKBH5 and FTO Regulate the Translation of ATF4 mRNA in Sorafenib-Treated Hepatocarcinoma Cells

**DOI:** 10.3390/biom14080932

**Published:** 2024-08-01

**Authors:** Pauline Adjibade, Sergio Di-Marco, Imed-Eddine Gallouzi, Rachid Mazroui

**Affiliations:** 1 Centre de Recherche du CHU de Québec-Université Laval, Axe Oncologie, Département de Biologie Moléculaire, Biochimie Médicale et Pathologie, Faculté de Médecine, Université Laval, Québec, QC G1V 0A6, Canada; pauline.adjibade.1@ulaval.ca; 2 KAUST Smart-Health Initiative (KSHI) and Biological and Environmental Science and Engineering (BESE) Division, King Abdullah University of Science and Technology (KAUST), Jeddah 21589, Saudi Arabia; sergio.dimarco@kaust.edu.sa (S.D.-M.); gallouzi.imed@kaust.edu.sa (I.-E.G.); 3 Department of Biochemistry, McGill University, Montreal, QC H3G 1Y6, Canada; 4 Rosalind & Morris Goodman Cancer Institute, McGill University, Montreal, QC H3A 1A3, Canada

**Keywords:** stress response, translation regulation, ALKBH5, FTO, RNA methylation

## Abstract

Translation is one of the main gene expression steps targeted by cellular stress, commonly referred to as translational stress, which includes treatment with anticancer drugs. While translational stress blocks the translation initiation of bulk mRNAs, it nonetheless activates the translation of specific mRNAs known as short upstream open reading frames (uORFs)-mRNAs. Among these, the ATF4 mRNA encodes a transcription factor that reprograms gene expression in cells responding to various stresses. Although the stress-induced translation of the ATF4 mRNA relies on the presence of uORFs (upstream to the main ATF4 ORF), the mechanisms mediating this effect, particularly during chemoresistance, remain elusive. Here, we report that ALKBH5 (AlkB Homolog 5) and FTO (FTO: Fat mass and obesity-associated protein), the two RNA demethylating enzymes, promote the translation of ATF4 mRNA in a transformed liver cell line (Hep3B) treated with the chemotherapeutic drug sorafenib. Using the in vitro luciferase reporter translational assay, we found that depletion of both enzymes reduced the translation of the reporter ATF4 mRNA upon drug treatment. Consistently, depletion of either protein abrogates the loading of the ATF3 mRNA into translating ribosomes as assessed by polyribosome assays coupled to RT-qPCR. Collectively, these results indicate that the ALKBH5 and FTO-mediated translation of the ATF4 mRNA is regulated at its initiation step. Using in vitro methylation assays, we found that ALKBH5 is required for the inhibition of the methylation of a reporter ATF4 mRNA at a conserved adenosine (A235) site located at its uORF2, suggesting that ALKBH5-mediated translation of ATF4 mRNA involves demethylation of its A235. Preventing methylation of A235 by introducing an A/G mutation into an ATF4 mRNA reporter renders its translation insensitive to ALKBH5 depletion, supporting the role of ALKBH5 demethylation activity in translation. Finally, targeting either ALKBH5 or FTO sensitizes Hep3B to sorafenib-induced cell death, contributing to their resistance. In summary, our data show that ALKBH5 and FTO are novel factors that promote resistance to sorafenib treatment, in part by mediating the translation of ATF4 mRNA.

## 1. Introduction

Gene expression reprogramming is fundamental for cell growth, proliferation, and differentiation. It is also critical for cell adaptation to stress and survival, involving mRNA translational regulatory pathways [1,2]. Among these, phosphorylation of the translation initiation factor eIF2α is the main pathway that is induced in cells exposed to various stresses, including endoplasmic reticulum (ER) stress, oxidative stress, nutrient deprivation, hypoxia, and cancer drug treatment [3,4]. The phosphorylation of eIF2α (p-eIF2α) causes a global inhibition of translation by stalling translation initiation complexes onto mRNAs. The resulting accumulation of untranslated mRNAs creates a microenvironment known as stress granules (SG), concentrating specific RNA-binding proteins, RNA, and translation initiation factors, limiting their translational activity, and further contributing to the p-eIF2α-mediated inhibition of translation. However, although p-eIF2α blocks the translation of bulk mRNAs, it allows the translation of a specific class of mRNAs known as short upstream open reading frames (uORFs) mRNAs [1,2]. The mechanisms responsible for the p-eIF2α-mediated translation of these messages, however, remain elusive.

ATF4 (Activating transcription factor) is a critical stress-induced factor whose expression relies on p-eIF2α. ATF4 mRNA has been widely used as a p-eIF2α-target paradigm, establishing that p-eIF2α-mediated translation occurs via a specialized mode of translation initiation relying on the presence of short, inhibitory uORFs at 5′untranslated region (UTR) of mRNAs [4]. Under normal cell growth conditions (where eIF2α is in an unphosphorylated state), the translation at uORFs prevents ribosomes from translating the overlapping ATF4 ORF [1,5]. Upon exposure to stress, the phosphorylation of eIF2α allows ribosomes to bypass the inhibitory uORFs, thus initiating translation of the ATF4 mRNA. The encoded ATF4 protein, consequently, activates transcription of downstream genes encoding either apoptotic (e.g., ATF3 and CHOP) or survival (e.g., chaperones, antioxidants, and amino acid synthases) associated factors [6,7]. These opposite functions of ATF4 in the cellular stress response depend largely on its expression level linked to its translational regulation by p-eIF2α. In cancer cells, the level of *ATF4* mRNA translation induced by p-eIF2α is critical to promote either cell death [6] or survival [7] to ER stress generated upon treatment with genotoxic drugs. The mechanisms modulating the differential ability of ATF4 to promote the death or survival of these cells, however, are poorly defined.

The N6-methyladenosine (m^6^A) is a key chemical modification in RNA regulating multiple RNA-based processes and was recently reported as critical for stress- and cancer-regulated RNA metabolism, including translation [8]. This m^6^A modification usually occurs in the consensus [G/A m^6^AC] sequence and is regulated by both the m^6^A methyltransferase complex and the two demethylases, ALKBH5 and FTO [9]. Both demethylases have emerged as potential promoters of cancer by regulating the stability and/or the translation of messages in both the nucleus and cytoplasm of cells [10]. In particular, ALKBH5 was shown to drive ATF4 mRNA translation in amino acids-deprived mouse embryonic fibroblasts (MEF) [11]. The underlying mechanism involves cytoplasmic demethylation of a specific and conserved m^6^A (A225) located at uORF2 of the *ATF4* mRNA. This demethylation event forces the ribosomes to bypass the translation initiation codon of uORF2, allowing the selection of the downstream main translation initiation codon for ATF4 translation. FTO was similarly shown to promote ATF4 expression in amino acids-deprived MEFs [11]. However, whether ALKBH5 or FTO are required for ATF4 mRNA translation in chemotherapeutic-treated cancer cells to promote resistance is still unknown.

We have previously shown that treatment of hepatocarcinoma cell lines (HCC), including Hep3B with the chemotherapeutic drug sorafenib (SOR) induces ER stress characterized by the phosphorylation of eIF2α and the downstream induction of ATF4 mRNA translation [12]. We further showed that preventing this SOR-induced ATF4 expression [12,13] significantly sensitizes HCC to treatment. Here, we report that ALKBH5 and FTO are novel polyribosome-associated proteins that drive ATF4 mRNA translation in Hep3B cells treated with SOR, thus promoting their resistance.

## 2. Materials and Methods

### 2.1. Cell Lines and Cell Culture

Hepatocarcinoma Hep3B cells were previously described [12,13]. Hep3B and derivatives were maintained in Dulbecco’s modified Eagles’ medium (DMEM) (Wisent, Saint-Jean-Baptiste, QC, Canada) supplemented with 5% heat-inactivated fetal bovine serum (FBS; Wisent, Saint-Jean-Baptiste, QC, Canada), penicillin and streptomycin at 37 °C in 5% CO_2_.

### 2.2. shRNA and siRNA Experiments

shRNA-mediated stable depletion of ALKBH5 and FTO was obtained using lentiviral shRNA. shRNA targeting ALKBH5 was generated by ligation of oligonucleotides into the *Age*I and *Eco*RI restriction sites of pLKO.1 (Addgene, Watertown, MA, USA; plasmid #8453). Lentiviral-shRNA particles were generated by transfecting HEK 293T cells with 12 μg of pLJM1 vector containing the shRNA, 6 μg of psPAX2 packaging plasmid (Addgene plasmid #12260), and 2 μg pMD2.G envelope plasmid (Addgene plasmid #12259). The media was changed 16 h after transfection, and lentiviral particles were harvested 24 h later. Viral supernatant was filtered through 0.45 μm filters and supplemented with 8 μg/mL polybrene (Sigma, Oakville, ON, Canada). The supernatant was added to the cells for 24 h before the start of puromycin selection.

siRNAs were obtained from Dharmacon (Lafayette, CO, USA). siRNA transfections were performed using Hiperfect reagent (Qiagen,Toronto, ON, Canada) following the manufacturer’s protocol on cells at 50–60% confluency. Annealed duplexes were used at a final concentration of 10 nM. Forty-eight hours posttransfection, cells were treated with the same siRNA (5 nM) for an additional forty-eight hours before treatment.

The sequences of shRNA/siRNA used are:

siALKBH5 sense sequence (NM_017758.3): 5′-GCU GCA AGU UCC AGU UCA A-3′

shALKBH5 sense sequence (NM_017758.3): 5′-CGG CAG AGT TGT TCA GGT T-3′

shFTO sense sequence (NM_001080432.3): 5′-GCT GAG GCA GTT TTG GTT TCA-3′

### 2.3. Stable GFP Transfection

mEGFP and mEGFP-ALKBH5 sequences were first ligated into the *Age*I and *PST*I restriction sites of a pLJM1 plasmid (Addgene, Watertown, MA, USA). Lentiviral particles were generated by transfecting HEK 293T cells with 12 μg of pLJM1-mEGFP or pLJM-ALKBH5 plasmids with 6 μg of psPAX2 packaging plasmid and 2 μg of pMD2.G envelope plasmid (Addgene, Watertown, MA, USA). The medium was replaced 16 h after transfection; lentiviral particles were harvested twenty-four hours later, filtered through 0.45 μm filters, and supplemented with 8 μg/mL polybrene (Sigma, Oakville, ON, Canada). The collected viruses were added to Hep3B cells for twenty-four hours before Hep3B stably expressing mEGFP or mEGFP-ALKBH5 were then selected via puromycin resistance selection.

### 2.4. Drugs Treatment

Sorafenib was purchased from Selleck Chemicals (Houston, TX, USA). Sorafenib was dissolved in DMSO as a 10 mM stock solution, aliquoted, and stored at −80 °C. MTT reagent was purchased from Sigma (Oakville, ON, Canada) and dissolved in 1X PBS at a concentration of 5 mg/mL. For drug treatment, cells were plated to reach a confluency of ~80–90% on the day of the treatment. The media was changed two hours prior to treatment.

### 2.5. Antibodies

Anti-DDX3 and anti-tubulin antibodies were purchased from Abcam (Waltham, MA, USA). Anti-ATF4, anti-ALKBH5, anti-FTO, and anti-m^6^A antibodies were obtained from Proteintech (Rosemont, IL, USA). RPL22 and RPS14 (from Santa Cruz Biotechnology, Dallas, TX, USA) were kindly provided by Dr Tom Moss (Université Laval). anti-FMRP antibodies were previously described [14].

### 2.6. Poly(ribo)some Profiling and Analyses of Polysomal-Associated Protein and mRNAs

Polysomes were obtained as previously described [15]. Briefly, cells grown in 100-mm tissue culture plates (~80–90% confluence) were treated with sorafenib, then lysed with 1ml of polysomal buffer (20 mM Tris, pH 7.4, 150 mM NaCl, 1.25 mM MgCl_2_, 5 U/mL RNase inhibitor [Invitrogen, Waltham, MA, USA], protease inhibitor cocktail [Complete; Roche, Mississauga, ON, Canada], 1 mM DTT, and Nonidet P-40 at a final concentration of 1%). Extracts were clarified by centrifugation (13,500 rpm, 20 min, 4 °C), and the resulting cytoplasmic extracts were loaded on a 15–55% (*w*/*v*) linear sucrose gradient for sedimentation by ultracentrifugation (35,000 rpm, 2 h 30 min, 4 °C). RNA-proteins complexes of individual isolated fractions were ethanol precipitated. For protein analysis, proteins were resuspended in an SDS-PAGE sample buffer. For RNA analysis, precipitated complexes were resuspended, and fractions corresponding to monosomes light and heavy polysomes were pooled and processed for RNA extraction and analyzed by RT-qPCR as described below.

### 2.7. Biotinylated ATF4 RNA Reporter

In vitro, transcribed and biotinylated ATF4 reporter RNA was produced as follows. Briefly, PCR-amplified DNA templates encoding 5′UTR of ATF4 mRNA were used for in vitro *transcription* of ATF4 reporter mRNA using HiScribe T7 High Yield RNA Synthesis Kit (NEB, Whitby, ON, Canada) according to the manufacturer’s protocols. The transcription products were purified with Monarch RNA Cleanup Kit (NEB, Whitby, ON, Canada), followed by polyadenylation using Poly(U) Polymerase (NEB, Whitby, ON, Canada) with Bio-16-UTP (Invitrogen, Waltham, MA, USA) and purification with Monarch RNA Cleanup Kit (NEB, Whitby, ON, Canada). RNAs were then used for MeRIP assays.

### 2.8. Methylated RNA Immunoprecipitation (MeRIP) Assay

Total RNA was isolated from the Hep3B cells using Trizol reagent (Life Technology, Waltham, MA, USA). Then, RNA was incubated overnight with ProteinA Sepharose beads coupled with anti-m^6^A antibody (Proteintech, Rosemont, IL, USA) or anti-IgG antibody in 500 μL of IP buffer (10 mM Tris-HCl (pH 7.4); 150 mM NaCl; 0.1% NP-40 substitute,1 mM DDT, 8U/mL RNase inhibitor (Invitrogen, Waltham, MA, USA)) at 4 °C. After washes, the beads were incubated with proteinase K buffer (10 mM Tris-HCl (pH 7.4); 150 mM NaCl; 0.1% NP-40 substitute; 1 mM DDT, 8U/mL RNase inhibitor (Invitrogen, Waltham, MA, USA), 2 mg/mL Proteinase K and 0.1% SDS) at 55 °C for 30 min. Immunoprecipitated RNA was extracted, and the expression of related genes was detected via RT-qPCR.

For MeRIP assay using the biotinylated ATF4 RNA reporter, untreated and sorafenib-treated Hep3B were lysed in a lysis buffer (50 mM Tris-HCl (pH 7.4); 150 mM NaCl; 1 mM MgCl_2_; 0.5% NP-40 substitute; 0.25 mM PMSF; 1 mM DDT, 5 U/mL RNase inhibitor, EDTA-free protease inhibitor cocktail (Roche, Mississauga, ON, Canada)). After centrifugation, the supernatants were incubated with 5 µg of the biotinylated reporter overnight at 37 °C, followed by incubation with protein A beads coupled with anti-m^6^A antibody (Proteintech, Rosemont, IL, USA) or anti-IgG antibody at 4 °C for four hours. After incubation with proteinase K buffer (10 mM Tris-HCl (pH 7.4); 150 mM NaCl; 0.1% NP-40 substitute; 1 mM DDT, 8 U/mL RNase inhibitor, 2mg/mL Proteinase K and 0.1% SDS) at 55 °C for 30 min, methylated RNAs were extracted and then incubated with streptavidin-agarose beads (Thermo Scientific™, Waltham, MA, USA) during 2 h at 4 °C. Following extraction, the methylated reporter was analyzed by RT-qPCR.

### 2.9. DNA Transfection and Luciferase Reporter Assay

Hep3B were transfected with a mixture of pRL-TK-Renilla, and the p5′UTR-ATF4-FLuc (WT ATF4-FLuc) or p5′UTR-ATF4 A235G-FLuc (mut ATF4-FLuc) plasmids using a Transfection reagent kit (Qiagen, Toronto, ON, Canada) as we previously described [13]. p5′UTR-ATF4 A235G-FLuc reporter mutant was produced by NorClone (London, ON, Canada). Forty-eight hours later, cells were treated with Thapsigargin (100 nM, four hours), and cells were resuspended in a passive lysis buffer (Promega, Madison, WI, USA). Firefly and *Renilla* luciferase activities were measured using a dual-luciferase reporter assay system (#E1960; Promega, Madison, WI, USA) according to the manufacturer’s instructions. Relative values of firefly luciferase activities were normalized to *Renilla* luciferase control.

### 2.10. Quantitative Real-Time PCR Analysis

Total RNA was extracted with the Trizol reagent (Life Technology, Waltham, MA, USA), and polyribosomal RNA was prepared using phenol-chloroform extraction. RNA was then reverse transcribed using the Quantitect Reverse Transcriptase kit (Qiagen, Toronto, ON, Canada). Real-time PCR reactions were prepared using the Luna^®^ Universal qPCR Master Mix (NEB, Whitby, ON, Canada). Reactions were run, and data was analyzed using the QuantStudio^TM^ 7 Flex Real-time PCR system (Applied Biosystems, Waltham, MA, USA). Data were calculated using the 2^−ΔΔCt^ method.

The primer sequences are:

for ATF4 mRNA (NM_001675.4): 5′-CACTAGGTACCGCCAGAAGA-3′ (forward)

5′-AATCCGCCCTCTCTTTTAGA-3′ (reverse)

For ALKBH5 mRNA (NM_017758.3): 5′-AAGGTGGAGGAGGAAGAAGC-3′ (forward)

5′-AAACAAAAGGAGGGGCAACT-3′ (reverse)

For FTO mRNA (NM_001080432.3): 5′-ACTTGGCTCCCTTATCTGACC-3′ (forward)

5′-TGTGCAGTGTGAGAAAGGCTT-3′ (reverse)

for GAPDH mRNA (NM_001289745.2): 5′-ACCCACTCCTCCACCTTTG-3′(forward)

5′-CCACCACCCTGTTGCTGT-3′ (reverse)

For 18s rRNA (M10098.1): 5′-AAACGGCTACCACATCCAAG-3′ (forward)

5′-CCTCCAATGGATCCTCGTTA-3′ (reverse)

For ATF4 RNA reporter: 5′- CCCGCCCACAGATGTAGTTT-3′ (Forward)

5′-GGCACTGCTGCCCCTAATAC-3′ (reverse)

### 2.11. MTT Assay

For MTT assays, 10,000 cells/well in 96-well plates were cultured overnight and then treated with 10 μM sorafenib for twenty-four hours. At the end of treatment, cells were washed with PBS and 3-(4,5-dimethyl-2-thiazolyl)2,5-diphenyl-2-H-tetrazolium bromide (MTT, Sigma-Aldrich, Oakville, ON, Canada) solution (final concentration: 0.5 mg/mL) was added to each well. Following two hours of incubation, the reaction was stopped with 150 μL DMSO, and the cells were shaken for 5 min at room temperature. The absorbance of each well was measured at 560 nm, and the viability of cells was calculated.

### 2.12. Clonogenic Assay

After treatment with sorafenib (10 μM, twenty-four hours), cells were trypsinized, counted, replated in 6-well plates at 1000 cells/well, and incubated for 8–10 days. Cells are then washed with PBS and stained (0.1% (*w*/*v*) crystal violet in a 0.0037% (*v*/*v*) formaldehyde solution in PBS). Isolated colonies were counted to determine cell viability.

### 2.13. Immunofluorescence

Immunofluorescence experiments were performed as previously described [13]. Briefly, after treatment, cells were fixed with 3.7% paraformaldehyde at room temperature (20 min) and permeabilized with methanol for 15 min at −20 °C. The sample was blocked with 1% BSA (30 min) and incubated with primary antibodies diluted in PBS containing 1% BSA and 0.1% Tween-20 for two hours at room temperature. After being rinsed with 0.1% Tween-20-PBS (PBST), cells were then incubated with anti-mouse/anti-rabbit IgG (H ≤ L) secondary antibodies coupled to Alexa Fluor 488 or 594 (Life Technology, Waltham, MA, USA) diluted in PBST for one hour, washed, and then mounted. Immunostainings were visualized using an LSM 900 laser scanning confocal microscope (Zeiss, Toronto, ON, Canada) controlled with ZEN blue 3.5 software for image acquisition and analysis.

## 3. Results

### 3.1. Depletion of ALKBH5 or FTO Downregulates Sorafenib (SOR)-Induced Expression of ATF4 mRNA

**Figure 1:**
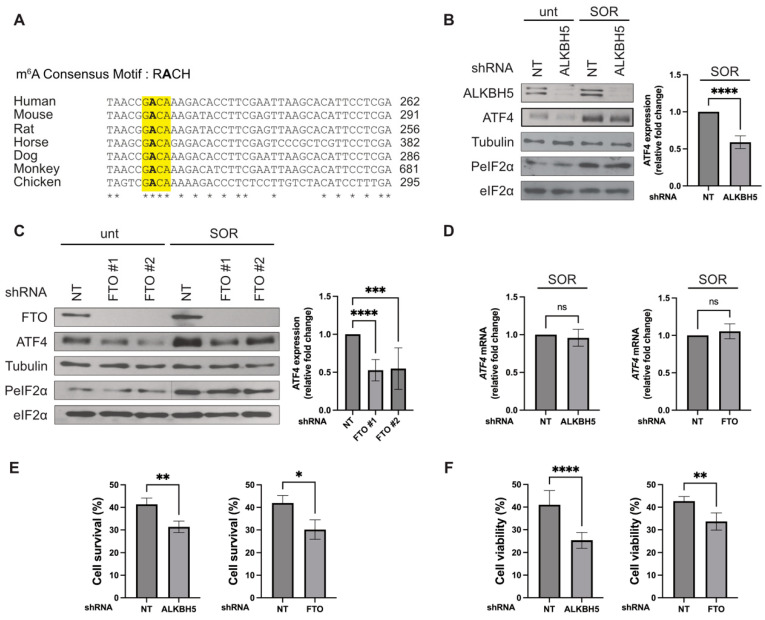
The RNA demethylases ALKBH5 and FTO are required for SOR-induced ATF4 expression. (**A**) Conserved consensus m^6^A site in the 5′UTR of ATF4 mRNA. R = A or G; H = A, C, or U, as described [18,19,20]. * corresponds to the conserved nucleotides, including the methylated adenosine in bold. (**B**–**D**) Hep3B was treated with 10 µM SOR for two hours. (**B**) Left panels: Cells were harvested and lysed, and protein extracts were analyzed by western blot for the expression of ALKBH5, ATF4, p-eIF2α, pan-eIF2α, and tubulin (Tub; loading control) using the corresponding antibodies. Right panel: The expression level of ATF4 was estimated by densitometry quantification of the film signal using Image Studio™ Lite Software (version 4.0.21) and standardized against total tubulin. **** *p* ≤ 0.0001 (Student’s *t*-test). (**C**) Left panels: Cells were harvested and lysed, and protein extracts were analyzed by western blot for the expression of the indicated proteins using the corresponding antibodies. Tubulin serves as a loading control. Right panel: The expression level of ATF4 was estimated by densitometry quantification of the film signal using Image Studio™ Lite Software and standardized against total tubulin. *** *p* ≤ 0.001; **** *p* ≤ 0.0001 (Student’s *t*-test). (**D**) Total RNA was isolated from harvested Hep3B, and the level of ATF4 mRNA relative to GAPDH mRNA was quantified by real-time q(RT)-PCR using the ΔΔCt method. The presented results are the mean of at least triplicate measurements, with error bars corresponding to the S.D. (**E**,**F**) Hep3B were treated with 10 µM SOR for twenty-four hours. (**E**) Clonogenic survival assays. After treatment, cells were trypsinized, counted, and seeded in the absence of the drug and incubated for 10 days. Populations > 20 cells were counted as one surviving colony. Data were calculated as the percentage of surviving colonies relative to the number found in plates corresponding to mock-depleted cell plates. Data shown are representative of at least 3 separate experiments, and values are given as mean ± SD. (Student’s *t*-test). ** *p* ≤ 0.01; * *p* ≤ 0.05. (**F**) Cell viability, assessed by MTT assay, shows the viability of Hep3B cells stably expressing shALKBH5 or shFTO after exposure to SOR for twenty-four hours. Data shown are representative of at least 3 separate experiments, and values are given as mean ± SD. (Student’s *t*-test). ** *p* ≤ 0.01; **** *p* ≤ 0.0001. Original images can be found in Supplementary Materials.

It was previously reported that mouse ALKBH5 drives ATF4 mRNA translation in amino acids-deprived mouse embryonic fibroblasts (MEF) [11]. The underlying mechanism involves the demethylation of a specific m^6^A225 residue located in the uORF2 of the ATF4 mRNA. This event allows ribosomes to select the main translation initiation codon for translation [11]. Mouse FTO was similarly shown to promote ATF4 expression in amino acids-deprived MEFs [11], potentially through a similar mechanism, though this was not investigated. Our in silico analysis of the uORF2 of human ATF4 mRNA confirmed that the m^6^A225 site is conserved in mammals (m^6^A235) (Figure 1A), which is consistent with previous m^6^A-seq data [16,17], identifying A235 as the potential methylation site in the 5′UTR of human ATF4 mRNA. We postulate that a similarly conserved epitranscriptomic mechanism involving m^6^A235 demethylating activity by ALKBH5 and/or FTO may contribute to the induction of ATF4 mRNA expression in cancer cells under stress conditions. We therefore assessed the importance of these demethylating enzymes in Hep3B liver cancer cells treated with SOR. Our previously published data and new data shown in Supplementary Figure S1A demonstrate that SOR induces the expression of ATF4 as early as 2 h posttreatment in a p-eIF2α [12] dependent manner, involving the activity of the *DEAD-box *RNA* helicase 3 *(DDX3) [13]. Here, we show that shRNA-mediated depletion of ALKBH5 or FTO (using a lentiviral system) significantly decreased ATF4 protein levels in SOR-treated Hep3B cancer cells (Figure 1B,C and Supplementary Figure S1). The reduced levels of ATF4 in ALKBH5 or FTO-depleted cells occur independently of an effect on eIF2α-mediated translation initiation since the treatment with SOR did not alter eIF2α phosphorylation (Figure 1B,C). Furthermore, we did not observe any differences in ATF4 mRNA expression under these conditions (Figure 1D), indicating that the SOR-induced expression of ATF4 is likely mediated at the translational level. Together, these results indicate that both ALKBH5 and FTO regulate the expression of ATF4 in liver cancer cells treated with SOR.

We have previously shown that preventing the SOR-induced expression of ATF4 (by either knocking down ATF4 or DDX3) results in the decreased survival of Hep3B cells [12,13]. We therefore tested if the knockdown of these demethylases has a similar effect on the survival of the Hep3B cells exposed to SOR. Hep3B cells depleted of ALKBH5 or FTO were treated with SOR and then collected and analyzed for survival by both clonogenic and MTT assays. Depletion of ALKBH5 and FTO significantly reduced the survival of SOR-treated Hep3B cells (Figure 1E,F), indicating that both ALKBH5 and FTO promote cell resistance to SOR. Collectively, these results indicate that ALKBH5 and FTO drive SOR resistance, possibly by promoting the induction of ATF4 expression.

### 3.2. ALKBH5 and FTO Are Polysome-Associated Proteins That Promote the Loading of ATF4 mRNA into Translating Ribosomes upon SOR Treatment

Both ALKBH5 and FTO have been described as nuclear proteins, though they have been implicated in cytoplasmic functions [9]. We thus tested if the ALKBH5- and FTO-mediated regulation of ATF4 expression during SOR treatment reflects a specific localization of the proteins. Due to the lack of anti-ALKBH5 suitable for immunofluorescence experiments, we used our Hep3B cells stably expressing GFP-ALKBH5. As expected, GFP-ALKBH5 distributes mainly in the nucleus of Hep3B cells (Supplementary Figure S2). We have previously shown that SOR treatment induces the formation of cytoplasmic stress granules (SG) that contain core components such as DDX3 and FMRP [12]. We did not detect GFP-ALKBH5 in SOR-induced SGs containing DDX3, which is consistent with previous data failing to detect this protein in SGs induced by arsenite in U2OS cells [16,21]. Similarly, we found FTO mainly localized to the nucleus of Hep3B cells (Supplementary Figure S2) [16]. In response to SOR treatment, FTO was not detected in FMRP- positive-SG (Supplementary Figure S2). Collectively, these results excluded an association of ALKBH5 and FTO with SOR-induced SGs, indicating that the FTO- and ALKBH5-induced expression of ATF4 occurs independently of the formation of these cytoplasmic entities.

**Figure 2:**
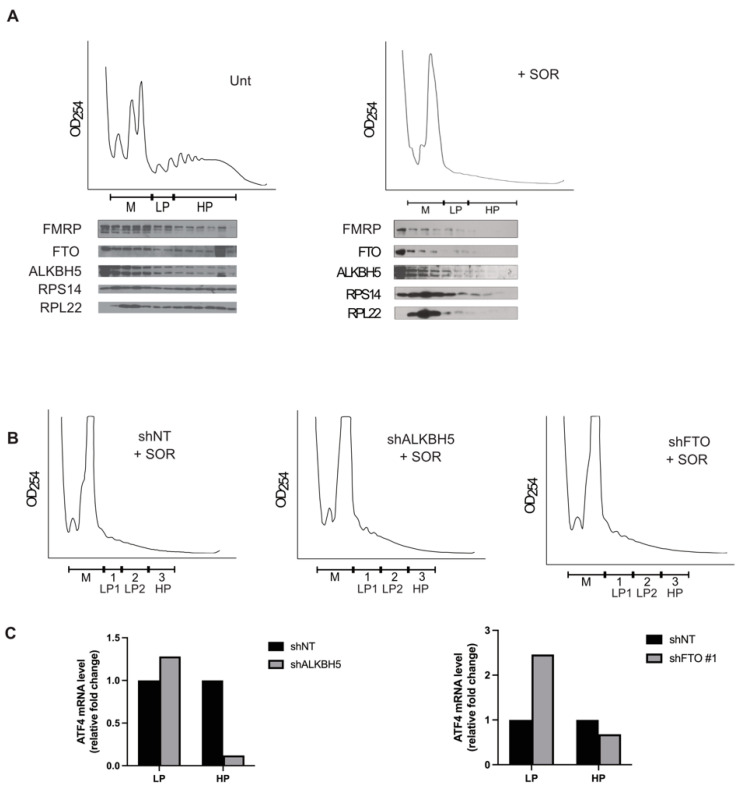
Both ALKBH5 and FTO are required for the association of ATF4 mRNA with polysomes in SOR-treated Hep3B. (**A**) Analysis of ALKBH5 and FTO association with polysomes. Top panels: cytoplasmic extracts prepared from either untreated or SOR-treated Hep3B were fractionated through 15–55% (*w*/*v*) sucrose density gradients, and their polysome profiles were monitored by measuring the OD254. M: monosomes. LP: light polysomes. HP: heavy polysomes. Bottom panels: Western blot analysis of the collected fractions for the distribution of FMRP, ALKBH5, and FTO using specific antibodies. RPS14 and RPL22 ribosomal proteins are used as controls for the integrity of the polysome profiles. The results are representative of two independent experiments. Original images can be found in Supplementary Materials. (**B**,**C**) Analysis of the ATF4 mRNA association with polysomes in the absence of the RNA demethylases ALKBH5 and FTO. Hep3B stably expressing shRNAs against ALKBH5 or FTO or a non-specific shRNA (NT) control were treated with SOR (10 µM, two hours) as above. Cytoplasmic extracts were fractionated through 15–55% sucrose gradients, and their polysome profiles were recorded as above. (**B**) Polysome profile of SOR-treated Hep3B shNT, shALKBH5 and shFTO. (**C**) RNA content was isolated from pooled LP and HP fractions, and associated ATF4 mRNA was quantified by RT-qPCR using the ΔΔCt method. ATF4 mRNA levels were normalized against 18S ribosomal RNA and expressed as indicated. The results are representative of two independent experiments.

The induction of ATF4 mRNA expression that occurs during treatment with SOR is mainly translational [12,13]. Our data showing that depleting either ALKBH5 or FTO prevents SOR-induced ATF4 protein levels (without any effect on its mRNA) suggested a possible role of these proteins in the translation of the ATF4 mRNA translation. To investigate this possibility, we first assessed ALKBH5 and FTO association with the translational machinery in untreated and SOR-treated Hep3B by performing polysome profiling analysis. Cytoplasmic extracts of untreated or SOR-treated Hep3B were processed through sucrose density gradients followed by separation of translation initiation complexes and translating polysomes fractions (Figure 2A). The polysome profiles were validated by western blot analysis of cell extracts of isolated fractions using anti-ribosomal RPL22, -RPS14, and -FMRP antibodies (Figure 2A, bottom panel). We observed that both ALKBH5 and FTO, under normal growth conditions, are distributed through the gradient (Figure 2A, left panel). This result suggests that the demethylases are associated with translation initiation complexes, sedimenting at the top of the gradients, and with translating polysomes sedimenting at the middle and bottom of the gradient, identifying ALKBH5 and FTO as polysomes-associated proteins. As we have previously described [12], treatment of Hep3B with SOR induces a significant collapse of polysomes (Figure 2A, right top panel), indicating a general inhibition of translation initiation. Despite the loss of the majority of polysomes in SOR-treated Hep3B, ALKBH5 and FTO are still detected, albeit slightly, in fractions corresponding to the residual translating polysomes (Figure 2A, right bottom panel), further supporting the possibility that ALKBH5 and FTO promote the induction of ATF4 mRNA translation upon SOR treatment. The association of ALKBH5 and FTO with polysomes also supports the role of these proteins in regulating the translation of ATF4 mRNA at the initiation step.

Targeting factors required for translation initiation generally result in the loss of the association of target mRNAs with translating polysomes. We have previously shown that depleting DDX3, a factor required for translation initiation of ATF4 mRNA, interferes with the association of the mRNA with polysomes [13]. We thus investigated if the depletion of ALKBH5 or FTO similarly affects the association of ATF4 mRNA with polysomes in SOR-treated Hep3B, reflecting a role of the proteins in the translation of this mRNA. In control experiments, depletion of ALKBH5 and FTO had no effect on the loss of polysomes induced by the treatment with SOR (Figure 2B), indicating that neither protein acts as a general translational regulator. Using RT-qPCR analysis of polysomes-associated mRNAs, we observed that depletion of ALKBH5 or FTO resulted in a significant decrease of the association of ATF4 mRNA with polysomes (Figure 2C, HP fraction), corroborating its translation repression. Together, these results indicate that ALKBH5 and FTO promote SOR-induced ATF4 mRNA translation, potentially at the initiation step.

### 3.3. ALKBH5 Interferes with the Methylation of ATF4 mRNA during SOR Treatment

**Figure 3:**
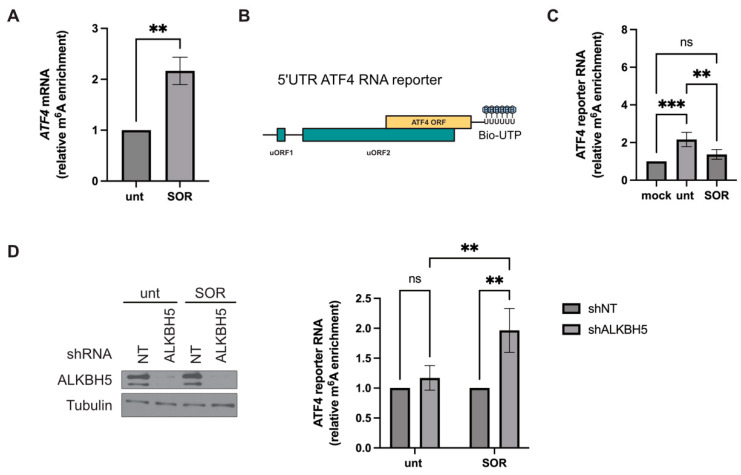
Role of ALKBH5 in ATF4 mRNA methylation level. (**A**) Methylated RNA immunoprecipitation (MeRIP)-qPCR analysis. Hep3B were treated with sorafenib (SOR; 10 μM) for two hours or left untreated (unt), lysed, and their extracts were subjected to MeRIP using m^6^A antibodies. Immunoprecipitated m^6^A RNAs are then quantified by RT-qPCR using oligos specific to the main ORF of ATF4 mRNA. The amounts of m^6^A ATF4 mRNA were normalized against IgG precipitate and then expressed relative to untreated conditions. ** *p* ≤ 0.01. (**B**) Schematic representation of the 5’UTR ATF4 RNA reporter. (**C**) MeRIP-qPCR analysis of m^6^A level of the biotinylated 5′UTR ATF4 RNA reporter incubated with protein extracts prepared from either untreated (unt) or sorafenib (SOR)-treated Hep3B. RNA is then subjected to MeRIP using m^6^A antibodies. Immunoprecipitated m^6^A RNA is then incubated with streptavidin-agarose beads to purify the biotinylated reporter RNA, which is quantified by RT-qPCR using oligos specific to the 5′end of ATF4 mRNA. The amounts of m^6^A ATF4 reporter RNA were normalized against IgG precipitate and then expressed relative to the mock condition. Data are representative of 3 separate experiments, and values are given as mean ± SD. ** *p* ≤ 0.01, *** *p* ≤ 0.001, ns: not significant. (**D**) MeRIP-qPCR analysis of m^6^A level of 5′UTR ATF4 RNA reporter incubated with proteins extracts from Hep3B stably expressing either a control shRNA (shNT) or shALKBH5 and treated with SOR (10 µM, 2 h). Depletion of ALKBH5 is validated by western blot using specific antibodies as described in Figure 1 (left panels). m^6^A methylated ATF4 reporter RNAs were isolated and quantified by RT-qPCR (right graphs) as above. The amounts of m^6^A ATF4 reporter RNA were normalized against IgG precipitate and then expressed relative to the shNT condition. Data are representative of 3 separate experiments, and values are given as mean ± SD. ** *p* ≤ 0.01, ns: not significant. Original images can be found in Supplementary Materials.

High-throughput sequencing of modified RNAs using approaches such as the methylated RNA immunoprecipitation (MeRIP) showed that m^6^A modifications are mainly enriched in 5′ UTRs, 3′ UTRs, and near-stop codons [19,22] and that the level of modified RNAs increases in response to stress such as oxidative stress [16] and heat shock [17]. In agreement, we found that treatment of Hep3B with SOR significantly increases the overall level of m^6^A of mRNAs as assessed in m^6^A dot blotting of Hep3B-purified poly(A) mRNAs using anti-m^6^A antibodies. We then performed MeRIP combined with RT-qPCR analyses to determine the m^6^A level of ATF4 mRNA in SOR-treated Hep3B. We observed ~2-fold increases in methylation of ATF4 mRNA in SOR-treated Hep3B as compared to untreated Hep3B (Figure 3A), indicating that SOR treatment induces a global methylation level of ATF4 mRNA, probably occurring at the multiple m^6^A sites found in its coding sequence, as previously reported in HEK293 or U2OS cells exposed to both oxidative stress and heat shock [16,17,23,24]. Similarly, several sites of ATF4 mRNA were shown to be methylated in amino acid-starved mouse embryonic fibroblasts. However, the conserved m^6^A225 located at the 5’UTR of ATF4 mRNA exhibited reduced methylation, an event required for its translation [17]. To investigate if this specific demethylation similarly occurs in SOR-treated Hep3B, we performed a modified MeRIP using an in vitro transcribed and biotinylated RNA reporter (Figure 3B,C). Briefly, the 5′UTR of human ATF4 mRNA was biotinylated in vitro and incubated without (mock) and with similar amounts of extracts prepared from either untreated- or SOR-treated Hep3B to allow methylation. The lysates were subjected to methylated RNA immunoprecipitation (MeRIP) using m^6^A antibodies and, as a negative control, IgG. Immunoprecipitated RNAs were then incubated with streptavidin-agarose beads to purify the biotinylated reporter RNA and quantified by RT-qPCR using oligos specific to the 5′end of ATF4 mRNA. The results show that incubation of the reporter RNA with Hep3B extracts efficiently induces methylation of its 5′UTR, possibly at the A235, which was, however, significantly reduced upon SOR treatment (Figure 3C). Thus, while incubation of the reporter ATF4 RNA with extracts of untreated Hep3B induces its methylation, most likely at the conserved A235 (Figure 1A), the lack of methylation of the reporter RNA in the extract of SOR-treated Hep3B supports our assumption that this treatment antagonizes m^6^A235 modification, potentially by activating a methylation antagonizing pathway, which may involve demethylases. Consistently, methylation of the reporter ATF4 mRNA was significantly enhanced upon incubation with extracts prepared from ALKBH5-depleted Hep3B and treated with SOR (Figure 3D), indicating that alteration of the methylation of the reporter mRNA in SOR-treated Hep3B occurs through a mechanism involving the activation of an ALKBH5 pathway. Control experiments (Figure 3D) showed that depletion of ALKBH5 does not affect the methylation status of the reporter mRNA incubated with the extracts from untreated Hep3B, indicating that ALKBH5 acts specifically upon SOR treatment for demethylation. Using similar MeRIP (Supplementary Figure S3), we did not observe a significant effect of FTO depletion on the methylation level of the reporter mRNA used in this assay (see discussion). Together, our data support the possibility that ALKBH5, via its demethylating activity, interferes with the methylation of the ATF4 RNA reporter at its A235 during stress, though we did not directly assess that demethylation.

### 3.4. ALKBH5-Mediated Demethylation of A235 of ATF4 mRNA Regulates Its SOR-Induced Translation

**Figure 4:**
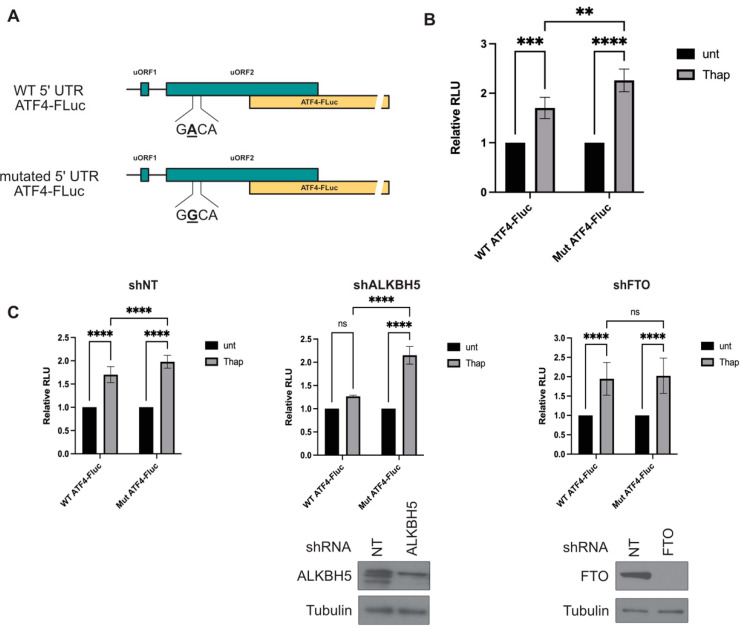
(**A**) Schematic representation of the human 5’UTR ATF4 Luciferase reporters (WT- and mut-ATF4 FLuc) consisting of the human WT- and A235G (mut)- ATF4 5′-UTR fused to Firefly luciferase (FLuc) gene. (**B**) Hep3B cells are co-transfected with either Fluc-expressing vectors or the control plasmid expressing *Renilla* luciferase (RLuc). Cells are then treated with Thapsigargin (thap) to induce translation of FLuc, the activity of which is measured in the cell extracts and expressed relative to RLuc. The relative values of firefly luciferase were shown as the average of three biological replicates. Error bars correspond to the S.D. ** *p* ≤ 0.01, *** *p* ≤ 0.001, **** *p* ≤ 0.0001. (**C**) Hep3B stably expressing either a control shRNA (shNT; left panels), shALKBH5 (middle panels), or shFTO (right panels) are co-transfected with a luciferase-expressing vector containing either the WT- or A235G (mut)- ATF4 5′-UTR fused to FLuc gene, and the control plasmid expressing RLuc. Cells are then treated with Thap to induce translation of FLuc, the activity of which is measured. The relative values of firefly luciferase were shown as the average of three biological replicates. Error bars correspond to the S.D. **** *p* ≤ 0.0001, ns: not significant. Original images can be found in Supplementary Materials.

To assess the assumption that ALKBH5-mediated demethylation of A235 of ATF4 mRNA regulates its translation, we devised the standard luciferase translational reporter assay that is widely used to assess the translation of uORFs-harboring mRNAs [13]. Briefly, mock- and ALKBH5-depleted Hep3B are co-transfected with either a luciferase-expressing vector containing the human ATF4 5′UTR fused to Firefy luciferase (FLuc) gene or its mutated version (mutATF4-FLuc) by introducing an A235G mutation (Figure 4A), together with the control plasmid expressing Renilla luciferase (RLuc). Cells are then treated with the drug to induce translation of FLuc, the activity of which is measured in the cell extracts and expressed relative to RLuc. As we previously described [13], SOR treatment does not induce efficient translation of the reporter in this assay, as compared to treatment with the canonical ER stress inducer Thapsigargin, precluding accurate and statistical quantification of FLuc level in SOR-treated cells. Thus, for these experiments, we used Thapsigargin, which we have shown to efficiently induce translation of both endogenous ATF4 and the FLuc reporter [13]. As expected, our control experiments showed that Thapsigargin treatment of either Hep3B (Figure 4B) or mock-depleted Hep3B (Figure 4C, left panels) significantly induced translation of both the WT- and mut- ATF4-FLuc. However, Thapsigargin-induced translation of the WT ATF4-Fluc, but not of the control mut ATF4-FLuc reporter, was prevented in ALKBH5-depleted Hep3B (Figure 4C, middle panels). In control experiments (Figure 4C; right panels), we found that FTO depletion similarly abrogates translation of the WT 5′UTR ATF4-Fluc and its mut(A235G)ATF4-FLuc mutant, indicating that FTO drives translation of ATF4 mRNA during drug treatment via alternative demethylation. Together, our data support that both ALKBH5 and FTO drive ATF4 mRNA translation through a mechanism involving demethylation of the ALKBH5-target A235 site.

## 4. Discussion

In this study, we found that both RNA demethylases ALKBH5 and FTO promote ATF4 mRNA expression in Hep3B, which is induced by SOR treatment. Our finding that either demethylase associates with polysomes and that their respective depletion abrogates the association of ATF4 mRNA with translating ribosomes in SOR-treated Hep3B supported the role of either demethylase in promoting the expression of ATF4 mRNA at the translational level. The findings that the level of methylation of the 5′UTR of ATF4 mRNA is increased upon depletion of ALKBH5 and that depletion of ALKBH5 abrogates the translation of the WT- but not the mut- ATF4 FLuc reporter are consistent with the role of the protein in antagonizing m^6^A, supporting a conserved RNA editing mechanism required for stress-induced ATF4 mRNA translation. Finally, our data showing that ALKBH5 and FTO are required for Hep3B to resist SOR treatment underscored the potential role of RNA demethylases in resistance to treatment, possibly by allowing translation of ATF4 mRNA.

Two studies reported the role of ALKBH5 in stress-mediated ATF4 mRNA expression, either by promoting its induction at the onset of stress or sustaining its expression during prolonged stress. In Zhou et al. study, mouse ALKBH5 was reported to be required to demethylate A225 of ATF4 mRNA and drive its translation in MEFs during the first 2 h of starvation [11], indicating that ALKBH5, and possibly FTO, are recruited to the ATF4 mRNA at the early phase of stress-inducing its demethylation and translation, though it remained unknown how this recruitment event occurs. Our data showing that 2 h of SOR treatment of Hep3B is sufficient to induce ATF4 mRNA translation, while this induction is prevented by depleting either ALKBH5 or FTO, support the role of demethylases at the early phase of stress required for the induction of ATF4 mRNA translation. The study of Mukhopadhyay et al. supported, however, a possible alternative ALKBH5-based regulatory mechanism of ATF4 mRNA translation [25]. In this mechanism, ALKBH5 is required to sustain the expression of ATF4 mRNA that occurs during persistent ER stress that is induced by prolonged (over 8 h) thapsigargin treatment of human HEK293T [25]. ALKBH5-mediated sustaining of ATF4 expression in thapsigargin-treated cells requires a significant increase in the expression level of ALKBH5 [25]. We did not observe, however, any change in the expression of ALKBH5 during SOR treatment (Supplementary Figure S1B), even during prolonged treatment, which is consistent with our assumption that ALKBH5 is required for the induction step of ATF4 translation during drug treatment. This is also consistent with our finding that depletion of ALKBH5 significantly abrogates translation of the FLuc reporter that occurs during acute thapsigargin treatment (Figure 4). Thus, the above studies, together with ours, support a translational role of ALKBH5 involving alternate stress- or cell-type-specific mechanisms, which may also depend on its expression level and binding partners.

Our MeRIP studies using the reporter biotinylated ATF4 mRNA and FLuc translational assays using the FLuc reporter harboring an A/G235 mutation are consistent with Zhou et al. showing that the A225 in mouse ATF4 mRNA, corresponding to the A235 in its human orthologue, is the main ALKBH5 target site for demethylation to promote ATF4 mRNA translation in human cells. In addition to m^6^A, both mouse and human ATF4 mRNA can be methylated at the first transcribed and conserved adenosine adjacent to the m^7^G cap through a modification called *N*^6^,2′-O-dimethyladenosine (m^6^Am) [25,26]. However, whether demethylation of m^6^Am is required for the induction of ATF4 mRNA translation upon stress remains unknown. Demethylation of m^6^Am is posed to be mediated mainly via FTO [27,28], regulating the stability of target mRNAs, with minimal effect on their translation [26]. Our depletion experiments revealed, however, a novel role of FTO in driving translation during stress. It is unlikely that FTO drives that translation by targeting the ALKBH5-A235 methylation site. First, in MeRIP using the biotinylated reporter ATF4 mRNA, which is uncapped and lacks the potential FTO-m^6^Am site, we found that FTO depletion had no significant effect on the methylation level of that reporter. This indicates that FTO is dispensable for demethylating A235 under our conditions. Second, in luciferase reporter assays, we found that depletion of FTO significantly abrogates translation of the capped ATF4-FLuc reporter harboring an A/G235 mutation, indicating that FTO drives translation of ATF4 mRNA by alternative demethylation, possibly at the m^6^Am, though we did not demonstrate this. Clearly, future studies are required to determine the contribution of m^6^Am demethylation in ATF4 mRNA translation.

We have previously shown that SOR treatment induced a dynamic repartition and localization of ATF4 mRNA [12]. We found that while a sub-fraction of ATF4 mRNA is associated with polysomes for translation, a significant fraction localizes into SG, potentially in a translation-repressed form [12]. At this stage, we do not know if the association of ATF4 mRNA with SG and polysomes is mediated by its methylation and demethylation at A235, respectively. Nevertheless, this assumption is consistent with reports showing that mRNAs harboring high levels of m^6^A, but not those lacking m^6^A are enriched in SG [16], which is also supported by data [16] showing an enrichment of m^6^A signal in SG induced in arsenite-treated cells, further supporting the possibility that modified RNAs are selectively sorted to SG, while demethylated mRNAs may be preferentially translated. In any case, future studies validating the role of ALKBH5 and FTO in demethylating the ATF4 mRNA during SOR treatment, combined with experiments assessing the localization of reporter ATF4 mRNAs harboring mutations at the methylation sites in SOR-induced SG, should help understand the dynamic role of m^6^A in the repartition of ATF4 mRNA between SG and polysomes, regulating its translation. Future investigations of the dynamic role of this RNA editing in translational regulation during drug treatment is an emerging area of research with potential discoveries of fundamental chemoresistance mechanisms.

## 5. Conclusions

In conclusion, our data show that the translation of ATF4 mRNA is tightly regulated by its m^6^A dynamics in sorafenib-treated Hep3B. The expression level of ATF4 is generally maintained to be low but drastically increased upon stress. ATF4 expression was also shown to be substantially elevated during early embryogenesis and testis development [29], which is consistent with spermatogenesis defects observed in ATF4 null mice and resembling infertility phenotypes observed in ALKBH5-deficient mice [20]. Whether the molecular mechanisms underlying the physiological role of ALKBH5 in spermatogenesis and embryogenesis involve ATF4 mRNA translation via its demethylation is a possibility that warrants future investigations. On the other hand, the physiological role of FTO has been mainly described in somatic tissues, performing metabolic functions of adipose and muscle tissues [9]. Given that ATF4 is a direct regulator of the expression of metabolic genes, it is tempting to speculate a prominent role of that FTO-ATF4 axis in the control of metabolic functions, though this remains to be demonstrated. Our study suggesting a role of ALKBH5- and FTO-ATF4 axes in cancer resistance is also consistent with the emerging role of either demethylase in the development of various types of tumors, including breast, gastric, and glioblastoma [9]. Future preclinical studies, i.e., using inhibitors of both FTO and ALKBH5 [9], are thus needed to evaluate the role of the ALKBH5 FTO-ATF4 axis in the resistance of cancer cells to treatment.

## Data Availability

The original contributions presented in the study are included in the article/Supplementary Material, further inquiries can be directed to the corresponding author.

## Supplementary Materials

The following supporting information can be downloaded at: https://www.mdpi.com/article/10.3390/biom14080932/s1, Figure S1: ATF4 and ALKBH5 expression in SOR-treated Hep3B; Figure S2: Localisation studies; Figure S3: MeRIP assays.

## References

[1] Pakos-Zebrucka K., Koryga I., Mnich K., Ljujic M., Samali A., Gorman A.M. (2016). The Integrated Stress Response. EMBO Rep..

[2] Vattem K.M., Wek R.C. (2004). Reinitiation Involving Upstream ORFs Regulates ATF4 MRNA Translation in Mammalian Cells. Proc. Natl. Acad. Sci. USA.

[3] Holcik M., Sonenberg N. (2005). Translational Control in Stress and Apoptosis. Nat. Rev. Mol. Cell Biol..

[4] Wek R.C., Jiang H.-Y., Anthony T. (2006). Coping with Stress: EIF2 Kinases and Translational Control. Biochem. Soc. Trans..

[5] Wek R.C., Cavener D.R. (2007). Translational Control and the Unfolded Protein Response. Antioxid. Redox Signal.

[6] Singleton D.C., Harris A.L. (2012). Targeting the ATF4 Pathway in Cancer Therapy Targeting the ATF4 Pathway in Cancer Therapy. Expert Opin. Ther. Targets.

[7] Zhang Z., Yin J., Zhang C., Liang N., Bai N., Chang A., Liu Y., Li Z., Tan X., Li N. (2012). Activating Transcription Factor 4 Increases Chemotherapeutics Resistance of Human Hepatocellular Carcinoma. Cancer Biol. Ther..

[8] Zhao W., Qi X., Liu L., Liu Z., Ma S., Wu J. (2020). Epigenetic Regulation of M6A Modifications in Human Cancer. Mol. Ther. Nucleic Acids.

[9] Shen D., Wang B., Gao Y., Zhao L., Bi Y., Zhang J., Wang N., Kang H., Pang J., Liu Y. (2022). Detailed Resume of RNA M6A Demethylases. Acta Pharm. Sin. B.

[10] Chen X.Y., Zhang J., Zhu J.S. (2019). The Role of M6A RNA Methylation in Human Cancer. Mol. Cancer.

[11] Zhou J., Wan J., Shu X.E., Mao Y., Liu X.M., Yuan X., Zhang X., Hess M.E., Brüning J.C., Qian S.B. (2018). N6-Methyladenosine Guides MRNA Alternative Translation during Integrated Stress Response. Mol. Cell.

[12] Adjibade P., St-Sauveur V.G.V.G., Quevillon-huberdeau M., Fournier M.J., Savard A., Coudert L., Khandjian E.W., Mazroui R., Huberdeau M.Q., Fournier M.J. (2015). Sorafenib, a Multikinase Inhibitor, Induces Formation of Stress Granules in Hepatocarcinoma Cells. Oncotarget.

[13] Adjibade P., Grenier St-Sauveur V., Bergeman J., Huot M.E., Khandjian E.W., Mazroui R. (2017). DDX3 Regulates Endoplasmic Reticulum Stress-Induced ATF4 Expression. Sci. Rep..

[14] Fournier M.-J., Coudert L., Mellaoui S., Adjibade P., Gareau C., Côté M.-F., Sonenberg N., Gaudreault R.C., Mazroui R. (2013). Inactivation of the MTORC1-EIF4E Pathway Alters Stress Granules Formation. Mol. Cell. Biol..

[15] Coudert L., Adjibade P., Mazroui R. (2014). Analysis of Translation Initiation During Stress Conditions by Polysome Profiling. J. Vis. Exp..

[16] Anders M., Chelysheva I., Goebel I., Trenkner T., Zhou J., Mao Y., Verzini S., Qian S.B., Ignatova Z. (2018). Dynamic M6a Methylation Facilitates MRNA Triaging to Stress Granules. Life Sci. Alliance.

[17] Zhou J., Wan J., Gao X., Zhang X., Jaffrey S.R., Qian S.B. (2015). Dynamic M6A MRNA Methylation Directs Translational Control of Heat Shock Response. Nature.

[18] Dominissini D., Moshitch-Moshkovitz S., Schwartz S., Salmon-Divon M., Ungar L., Osenberg S., Cesarkas K., Jacob-Hirsch J., Amariglio N., Kupiec M. (2012). Topology of the Human and Mouse M6A RNA Methylomes Revealed by M6A-Seq. Nature.

[19] Meyer K.D., Saletore Y., Zumbo P., Elemento O., Mason C.E., Jaffrey S.R. (2012). Comprehensive Analysis of MRNA Methylation Reveals Enrichment in 3′ UTRs and near Stop Codons. Cell.

[20] Zheng G., Dahl J.A., Niu Y., Fedorcsak P., Huang C.M., Li C.J., Vågbø C.B., Shi Y., Wang W.L., Song S.H. (2013). ALKBH5 Is a Mammalian RNA Demethylase That Impacts RNA Metabolism and Mouse Fertility. Mol. Cell.

[21] Jain S., Wheeler J.R.R., Walters R.W.W., Agrawal A., Barsic A., Parker R. (2016). ATPase-Modulated Stress Granules Contain a Diverse Proteome and Substructure. Cell.

[22] Ke S., Alemu E.A., Mertens C., Gantman E.C., Fak J.J., Mele A., Haripal B., Zucker-Scharff I., Moore M.J., Park C.Y. (2015). A Majority of M6A Residues Are in the Last Exons, Allowing the Potential for 3′ UTR Regulation. Genes Dev..

[23] Xuan J., Chen L., Chen Z., Pang J., Huang J., Lin J., Zheng L., Li B., Qu L., Yang J. (2024). RMBase v3.0: Decode the Landscape, Mechanisms and Functions of RNA Modifications. Nucleic Acids Res..

[24] Sun W.J., Li J.H., Liu S., Wu J., Zhou H., Qu L.H., Yang J.H. (2016). RMBase: A Resource for Decoding the Landscape of RNA Modifications from High-Throughput Sequencing Data. Nucleic Acids Res..

[25] Mukhopadhyay S., Amodeo M.E., Lee A.S.Y. (2023). EIF3d Controls the Persistent Integrated Stress Response. Mol. Cell.

[26] Boulias K., Toczydłowska-Socha D., Hawley B.R., Liberman N., Takashima K., Zaccara S., Guez T., Vasseur J.J., Debart F., Aravind L. (2019). Identification of the M6Am Methyltransferase PCIF1 Reveals the Location and Functions of M6Am in the Transcriptome. Mol. Cell.

[27] Wei J., Liu F., Lu Z., Fei Q., Ai Y., He P.C., Shi H., Cui X., Su R., Klungland A. (2018). Differential M6A, M6Am, and M1A Demethylation Mediated by FTO in the Cell Nucleus and Cytoplasm. Mol. Cell.

[28] Mauer J., Luo X., Blanjoie A., Jiao X., Grozhik A.V., Patil D.P., Linder B., Pickering B.F., Vasseur J.J., Chen Q. (2017). Reversible Methylation of M6 Am in the 5′ Cap Controls MRNA Stability. Nature.

[29] Muir T., Wilson-Rawls J., Stevens J.D., Rawls A., Schweitzer R., Kang C., Skinner M.K. (2008). Integration of CREB and bHLH transcriptional signaling pathways through direct heterodimerization of the proteins: Role in muscle and testis development. Mol. Reprod. Dev..

