# Supplementary material for: The RNA Demethylases ALKBH5 and FTO Regulate the Translation of ATF4 mRNA in Sorafenib-Treated Hepatocarcinoma Cells
Source: Biomolecules. 2024 Aug 1;14(8):932. doi: 10.3390/biom14080932 (PMC11352178; doi:10.3390/biom14080932)
Supplement: Supplementary file 1 [file biomolecules-14-00932-s001.zip › biomolecules-3061153-supplementary figures.pdf]

Supplementary data

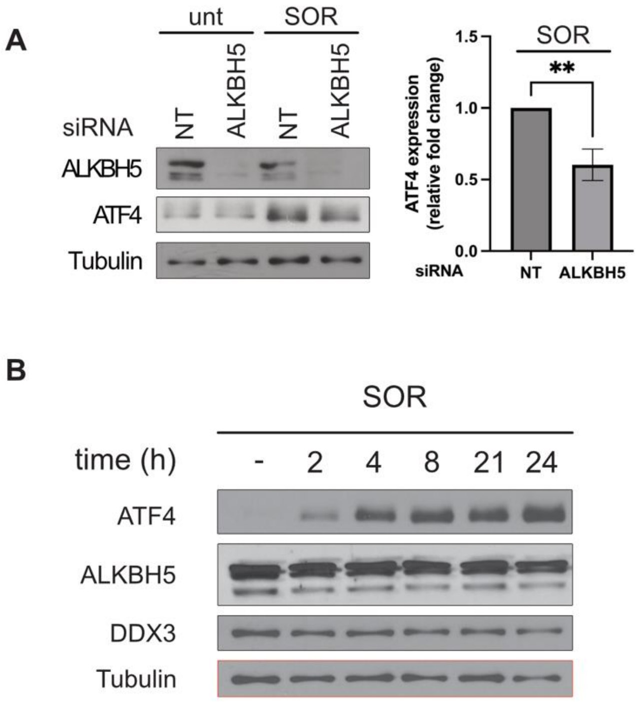

Supplementary Figure S1. (A) Hep3B were treated with ALKBH5 or control (NT) siRNAs for ninety-six hours and then incubated with 10 μM SOR for two hours. Left panel: Cells were harvested, lysed and protein extracts were analyzed by western blot for the expression of ATF4, ALKBH5 and tubulin (Tub; loading control) using the corresponding antibodies. Right panel: The expression level of ATF4 was estimated by densitometry quantification of the film signal using Image Studio™ Lite Software and standardized against total tubulin. $^{**}P \leq 0.01$ (Student's t-test). (B) Hep3B were treated with 10 μM SOR for the indicated times. Cells were collected, and their protein extracts were analyzed by western blot for the expression of ATF4, ALKBH5. DDX3 and tubulin served as loading controls.

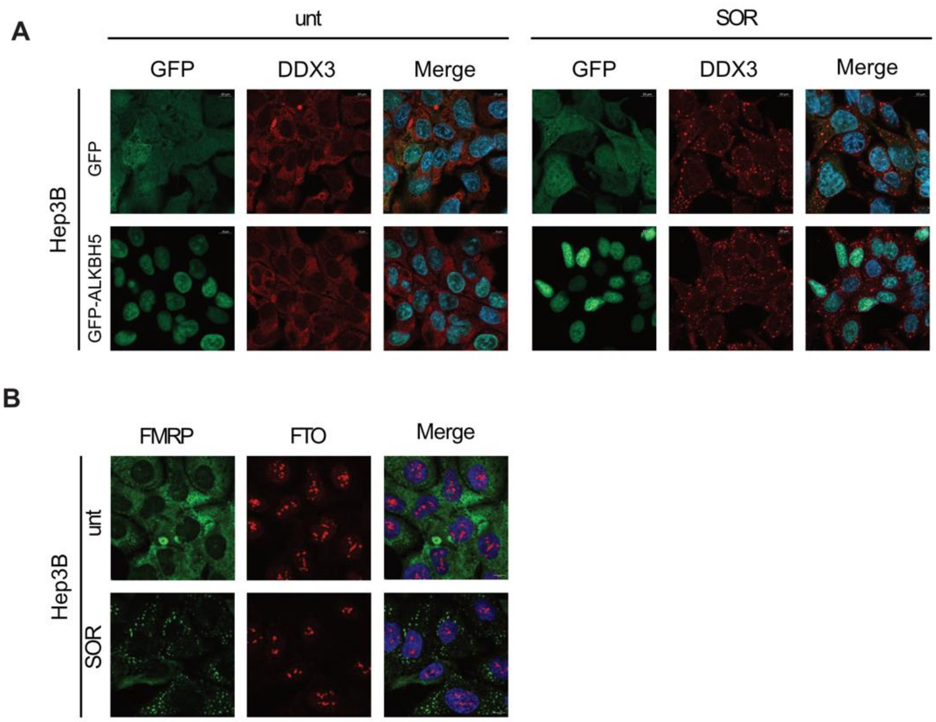

Supplementary Figure S2. Localisation studies. (A) Hep3B expressing GFP-ALKBH5 or GFP are treated with sorafenib (SOR; 10 μM) for two hours or left untreated (UNT). Cells are then fixed and processed for immunostaining using anti-DDX3 antibodies to detect SG. (B) Hep3B are treated with sorafenib (SOR; 10 μM) for two hours or left untreated (UNT). Cells are then fixed and processed for immunostaining using anti-FTO antibodies. Anti-FMRP are used to visualise SG.

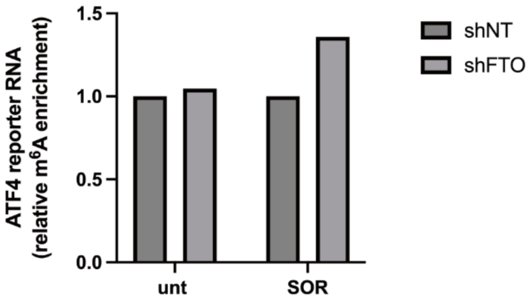

Supplementary Figure S3. MeRIP-qPCR analysis of m^6A level of the biotinylated 5'UTR ATF4 RNA reporter incubated with proteins extracts from Hep3B stably expressing either a control shRNA (shNT) or shFTO and treated with SOR (10 μM, two hours). m^6A methylated ATF4 reporter RNAs were isolated and quantified by RT-qPCR (right graphs) as described in figure 4. The amounts of m^6A ATF4 reporter RNA were normalized against IgG precipitate and then expressed relative to shNT condition.
