# Supplementary material for: The RNA Demethylases ALKBH5 and FTO Regulate the Translation of ATF4 mRNA in Sorafenib-Treated Hepatocarcinoma Cells
Source: Biomolecules. 2024 Aug 1;14(8):932. doi: 10.3390/biom14080932 (PMC11352178; doi:10.3390/biom14080932)
Supplement: Supplementary file 1 [file biomolecules-14-00932-s001.zip › Blots.pdf]

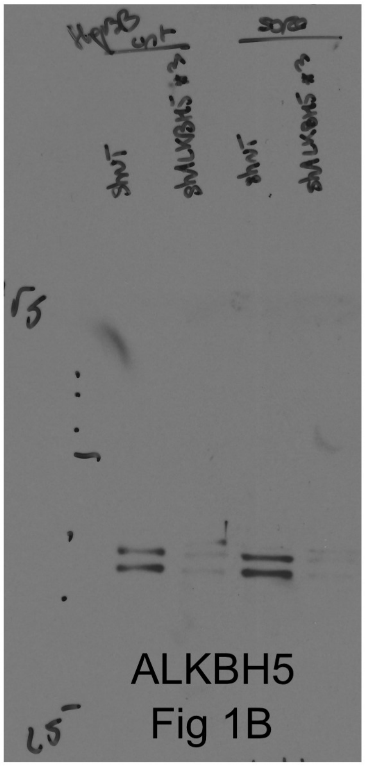

ALKBH5
Fig 1B

Fig 1B

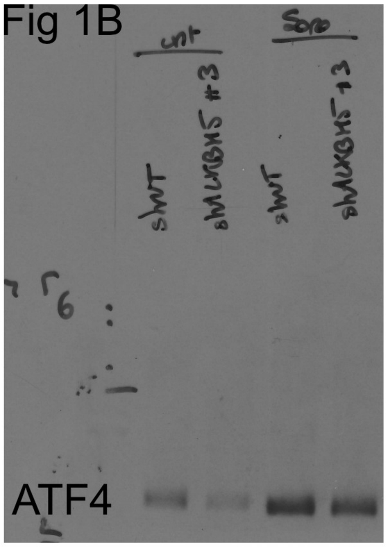

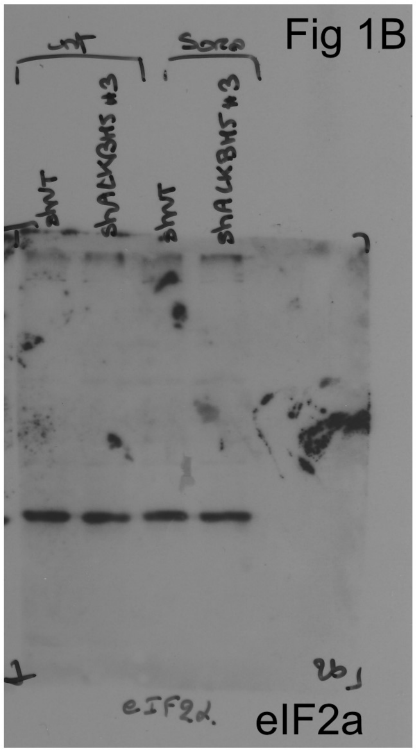
Fig 1B
Sora
shWT
shALKBH5 #3
shWT
shALKBH5 #3
eIF2α
eIF2a

Fig 1B

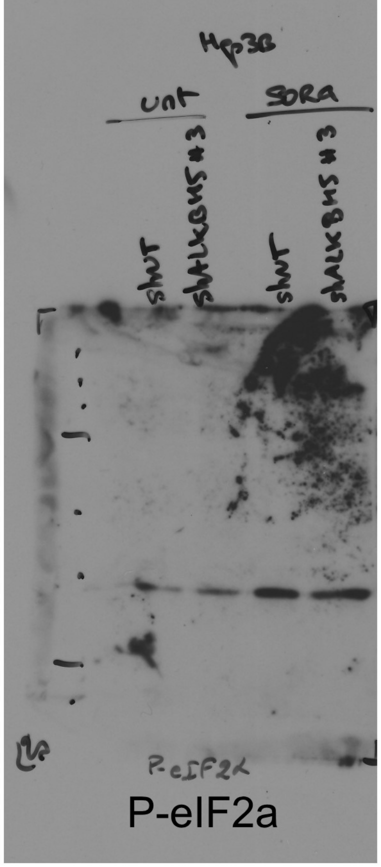

P-eIF2a

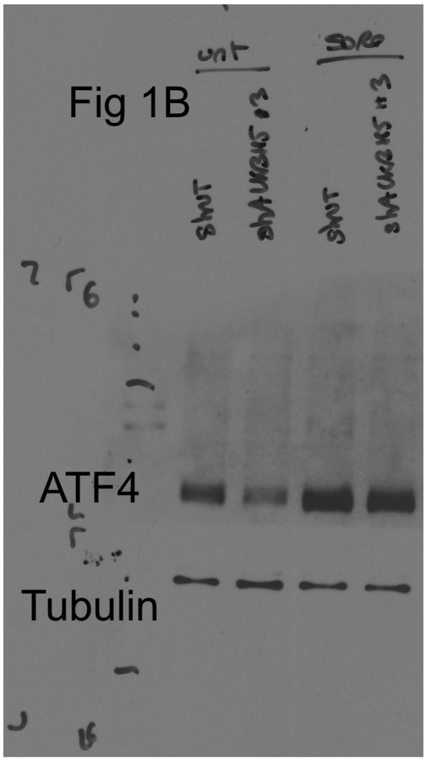
Fig 1B
ATF4
Tubulin

Fig 1C

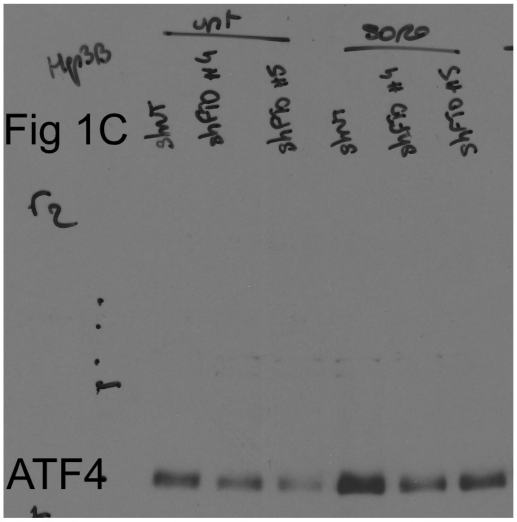

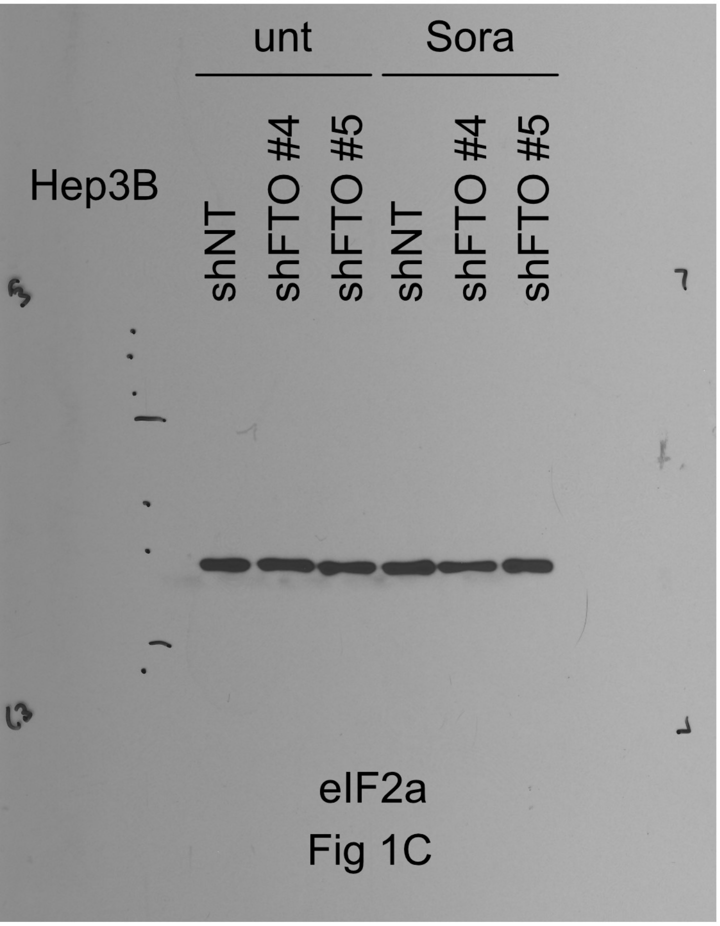

Fig 1C

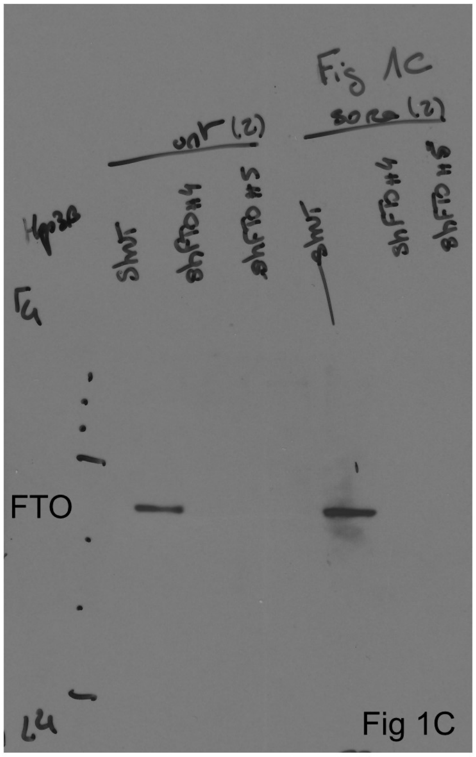
FTO
Fig 1C

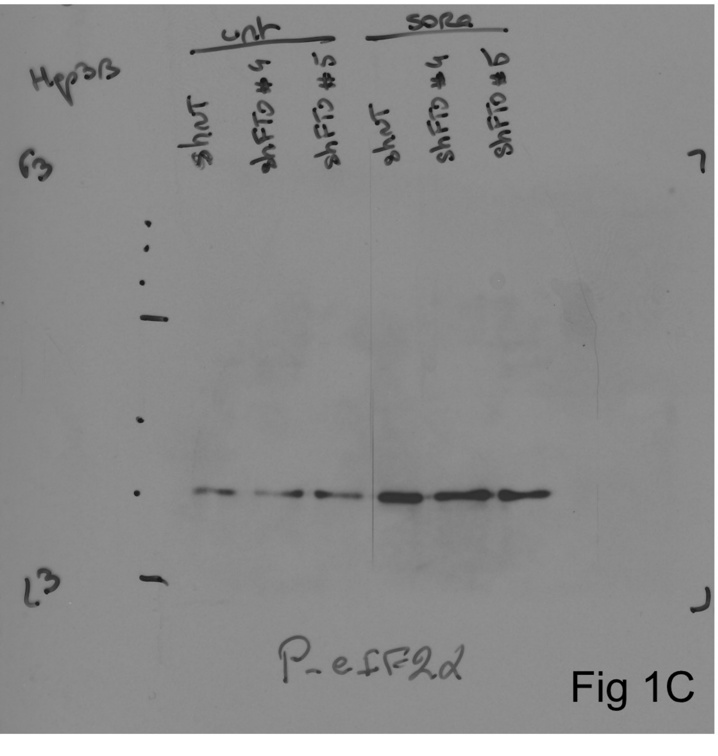
Hep3B
ctrl
sora
shNT
shFTO #4
shFTO #5
shNT
shFTO #4
shFTO #5
P-eIF2α

Fig 1C

Fig 1C

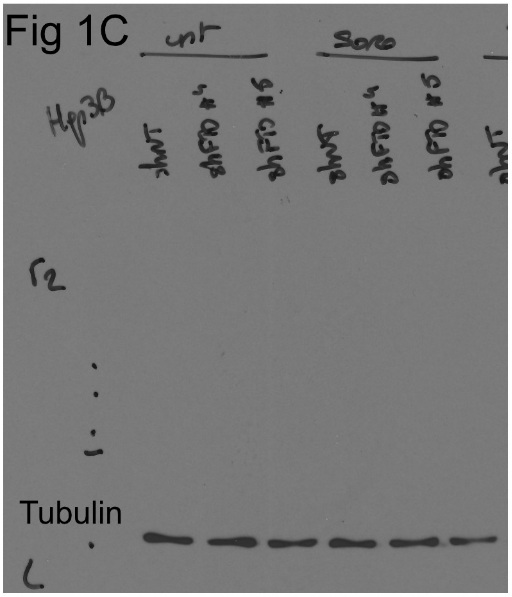

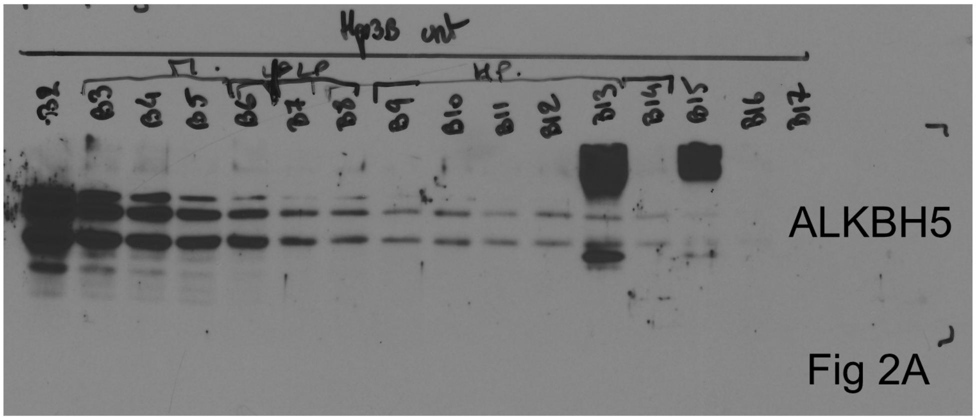

Fig 2A

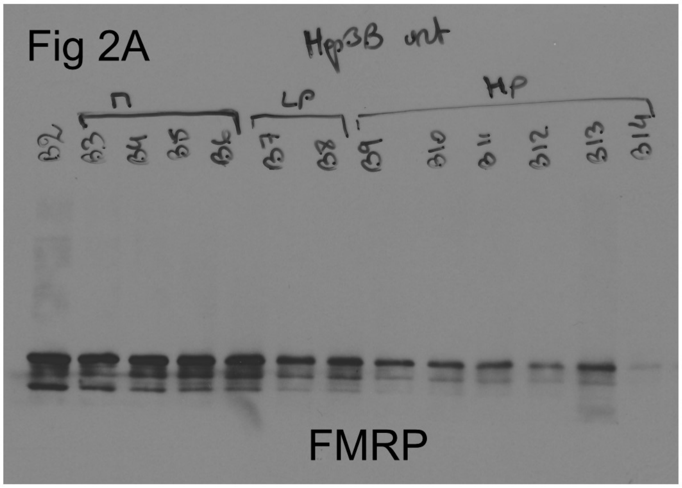
Fig 2A
FMRP

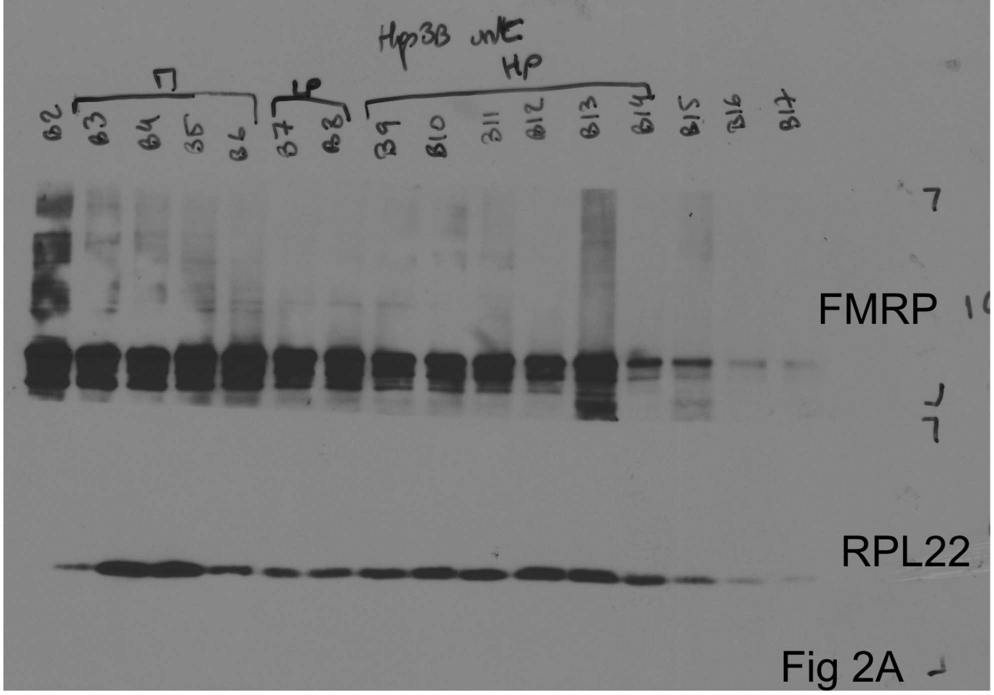
Hep3B untr
M
LP
HP
B2
B3
B4
B5
B6
B7
B8
B9
B10
B11
B12
B13
B14
B15
B16
B17
FMRP
RPL22
Fig 2A

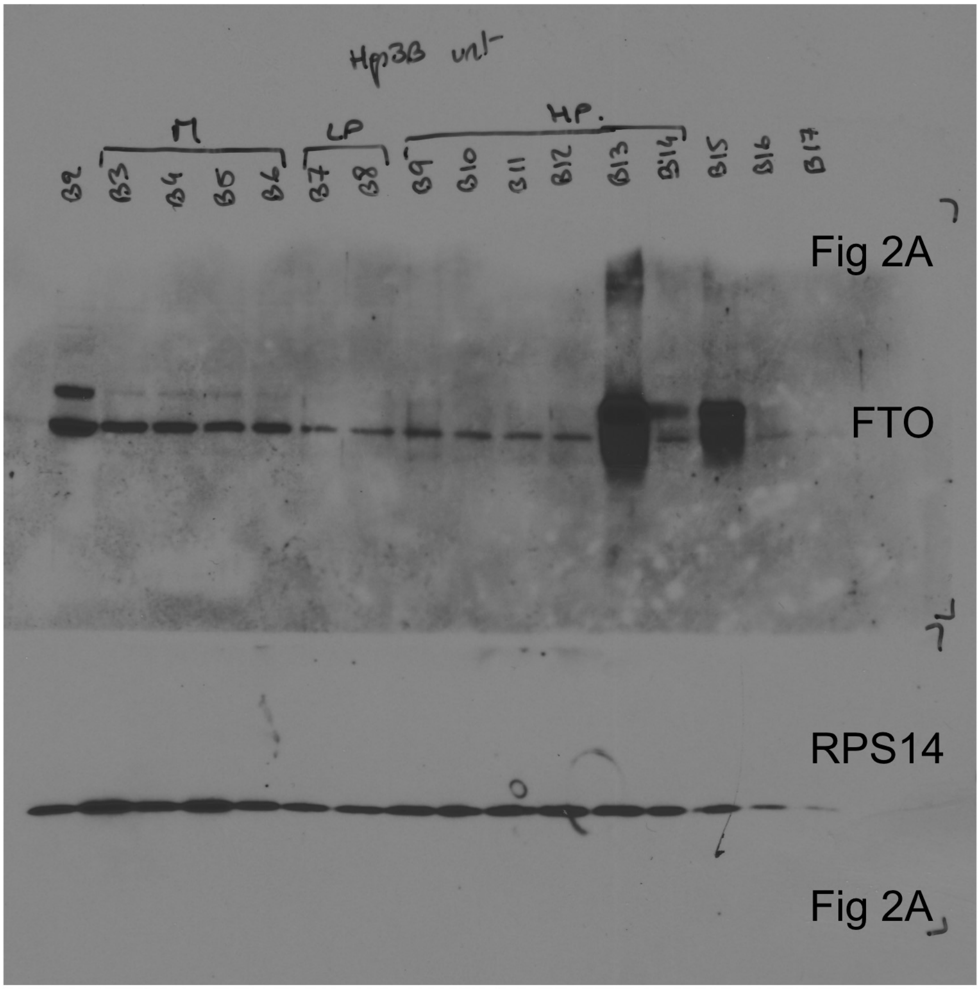
Hep3B untr
M
LP
HP.
B2
B3
B4
B5
B6
B7
B8
B9
B10
B11
B12
B13
B14
B15
B16
B17
Fig 2A
FTO
RPS14
Fig 2A

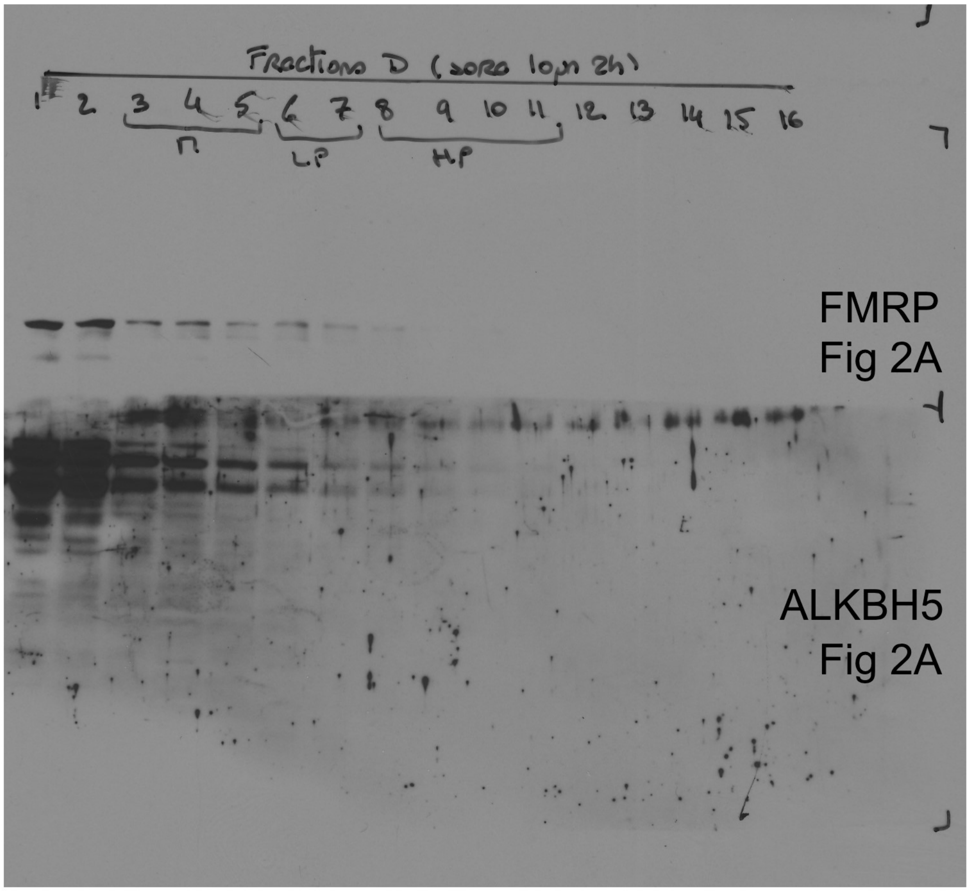

Fractions D (2020 10μn 2h)
1 2 3 4 5 6 7 8 9 10 11 12 13 14 15 16
LP
HP
FMRP
Fig 2A
ALKBH5
Fig 2A

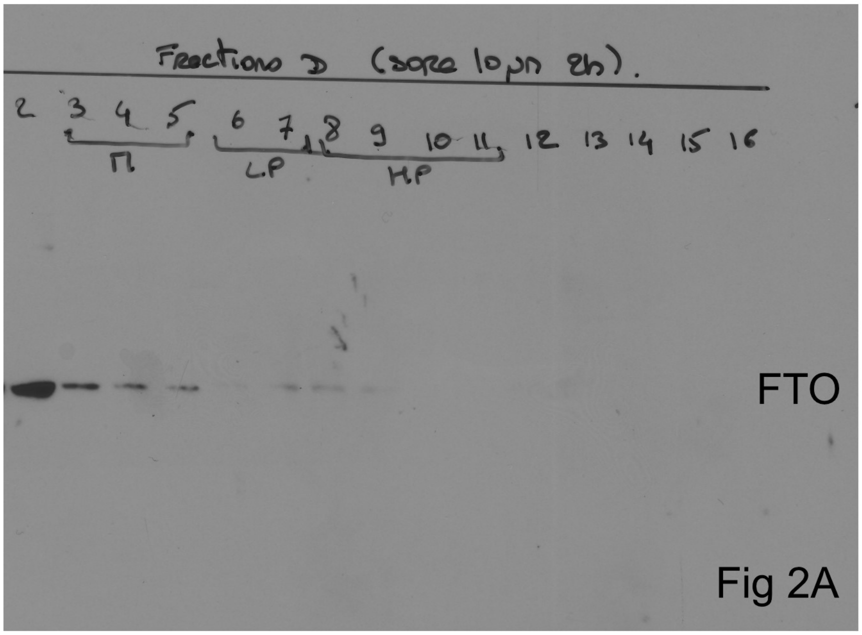

Fig 2A

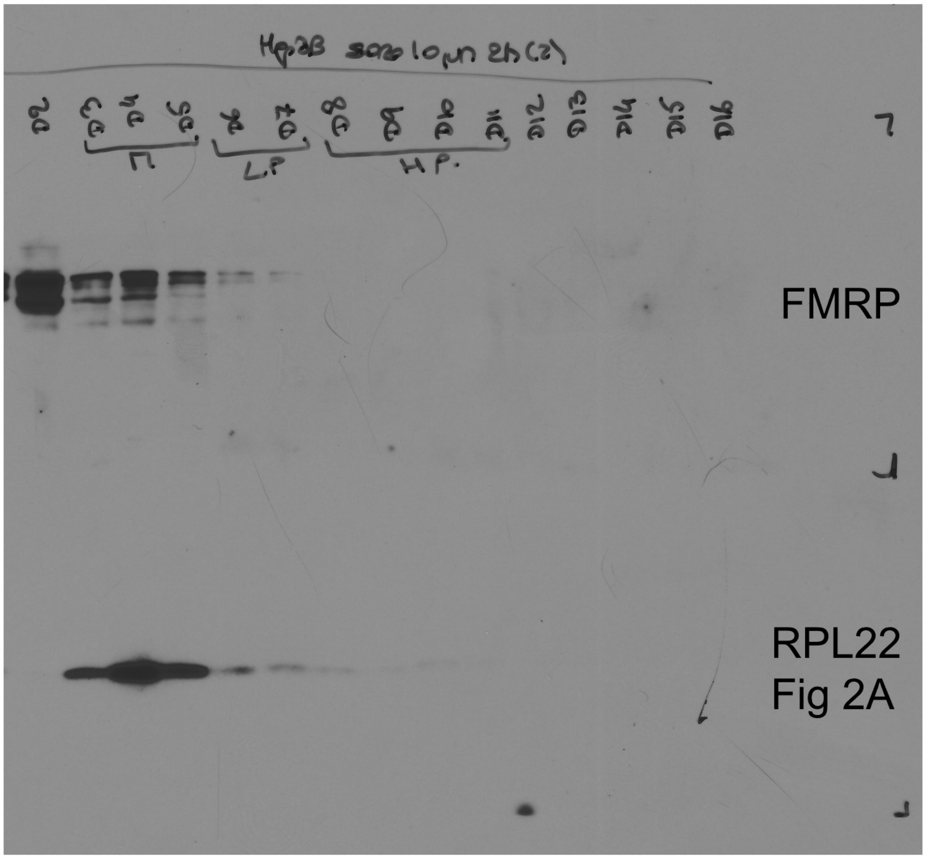

Fig 2A

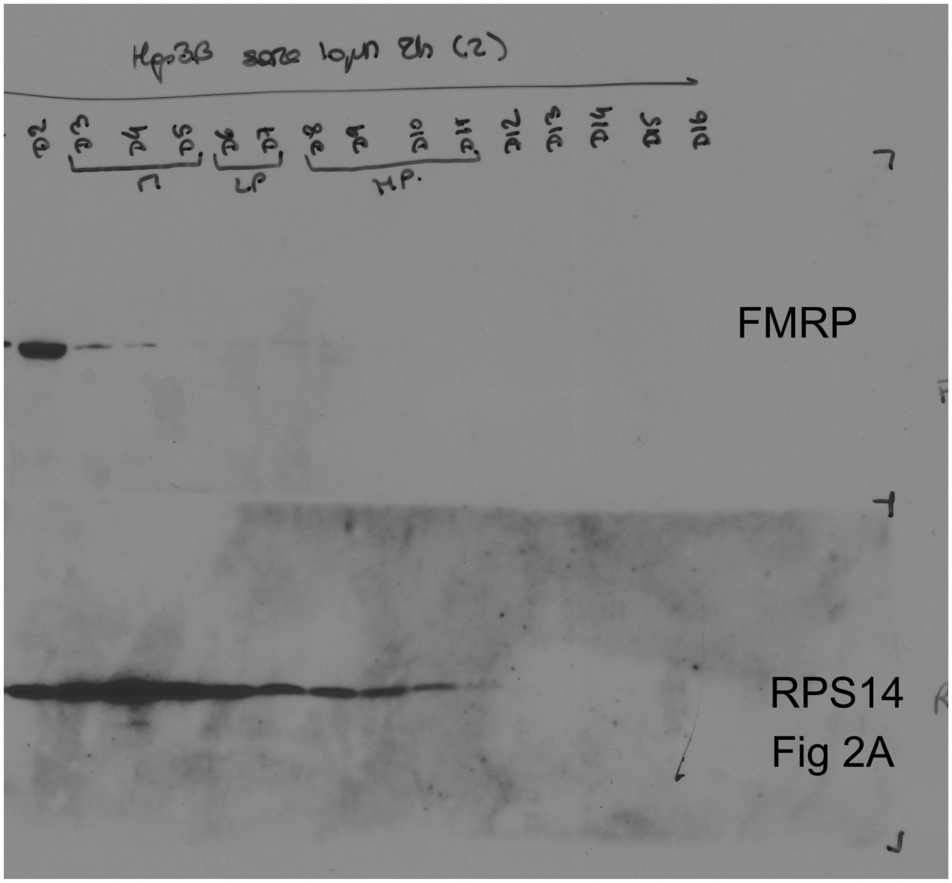

Fig 2A

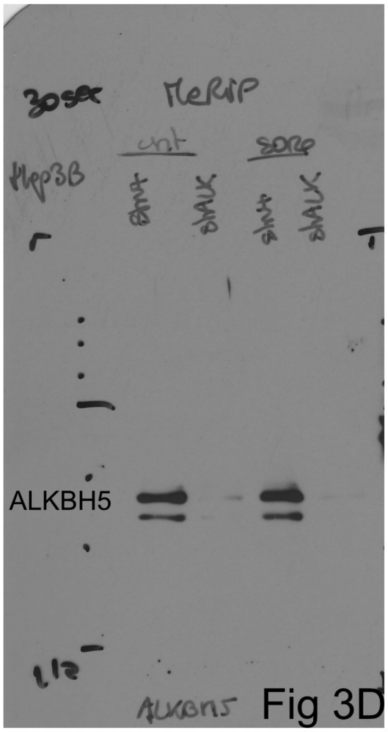

Fig 3D

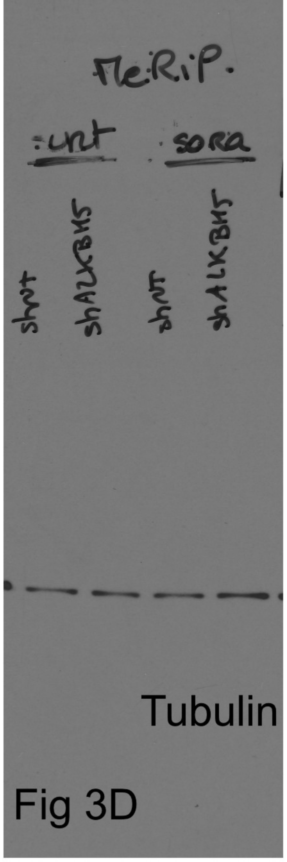

Fig 3D

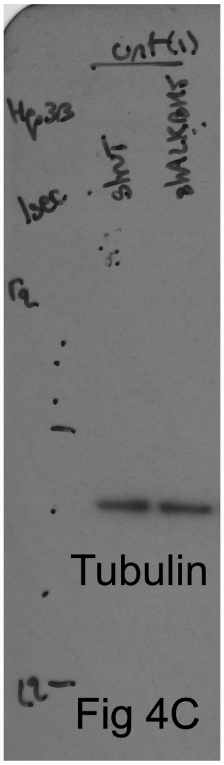
Tubulin
Fig 4C

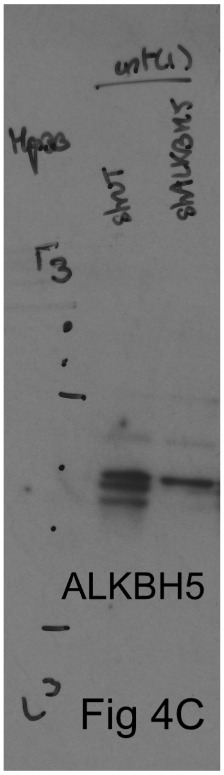
ALKBH5
Fig 4C

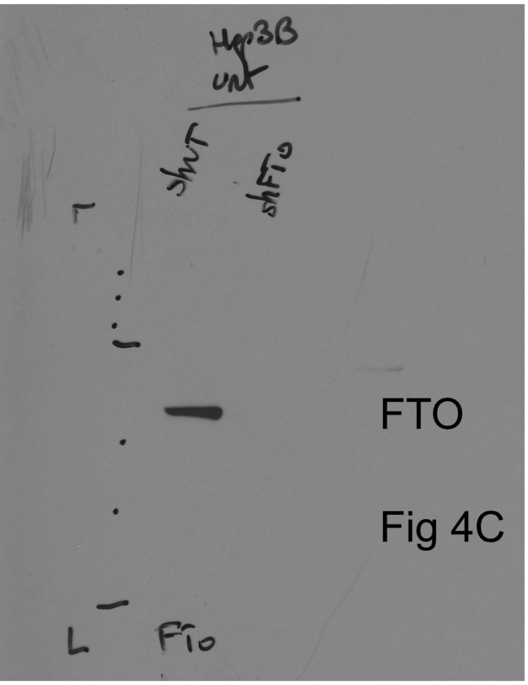

Fig 4C

Fig 4C

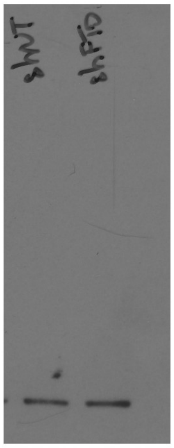

Tubulin

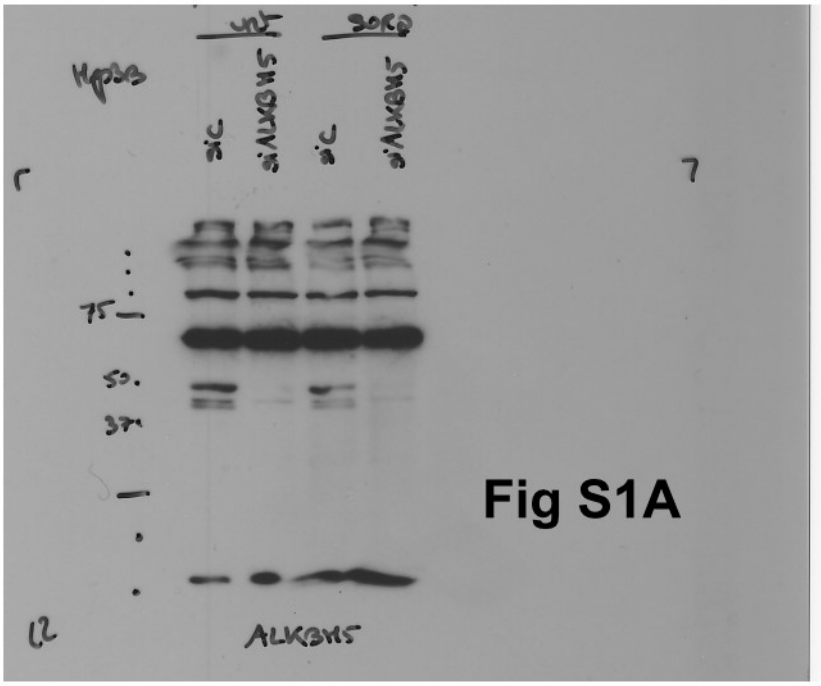

Fig S1A

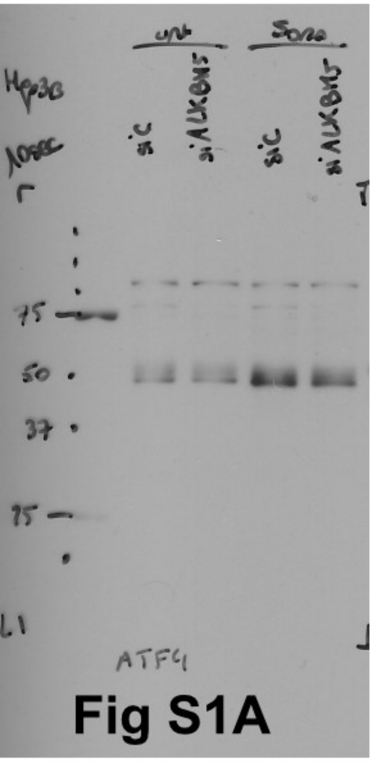

Fig S1A

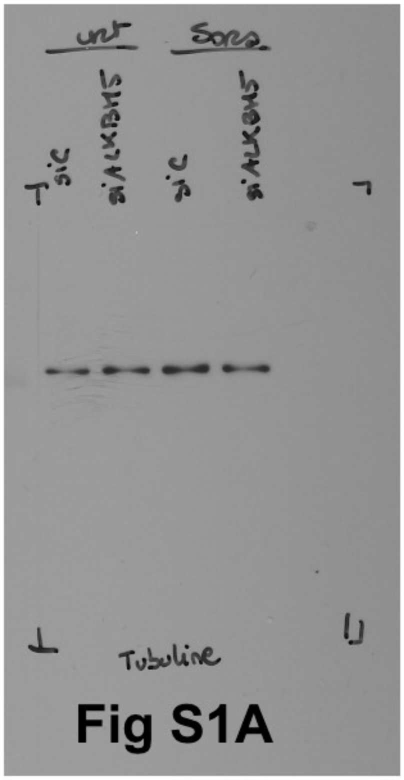

Fig S1A

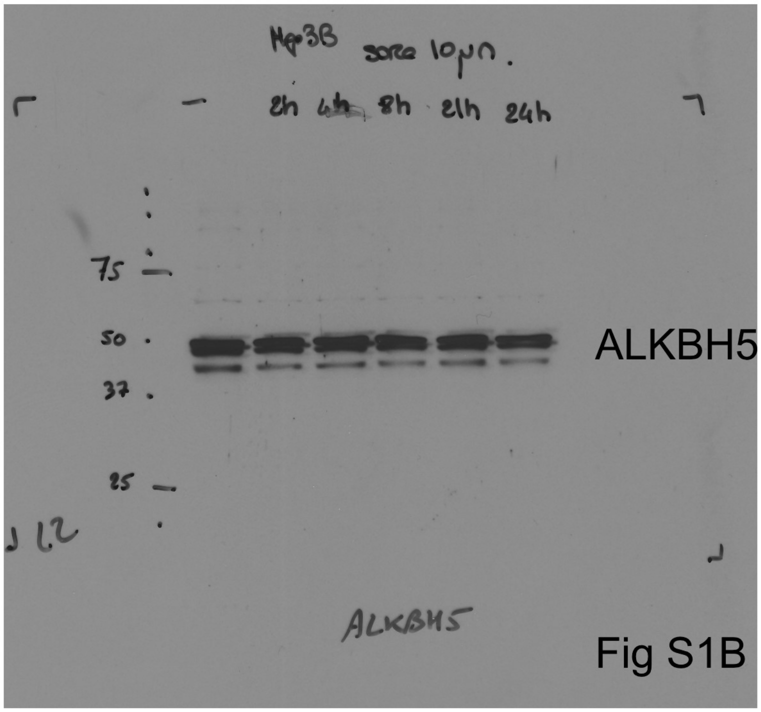

Fig S1B

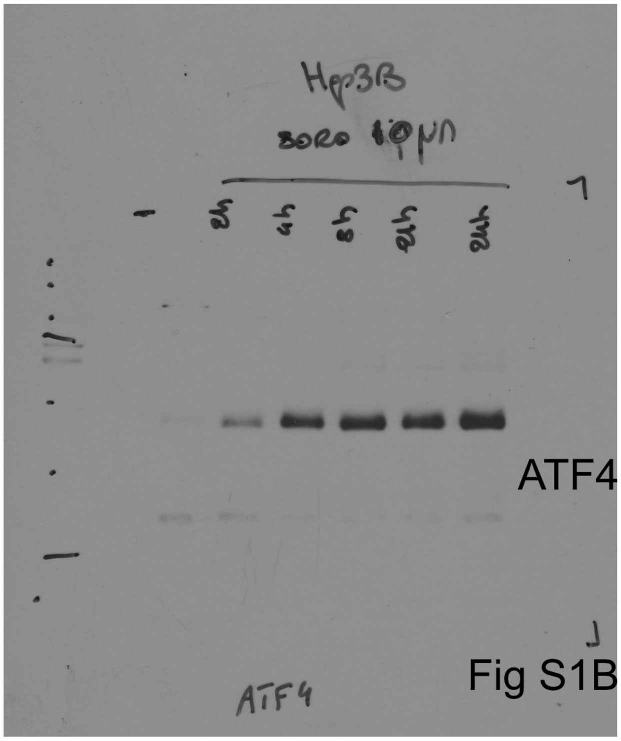

Fig S1B

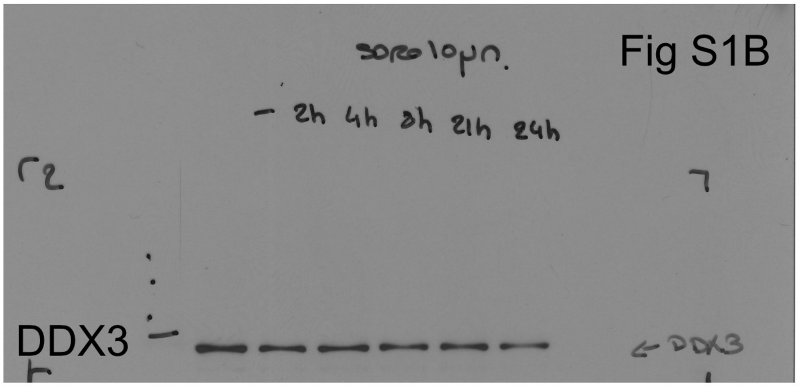

Fig S1B

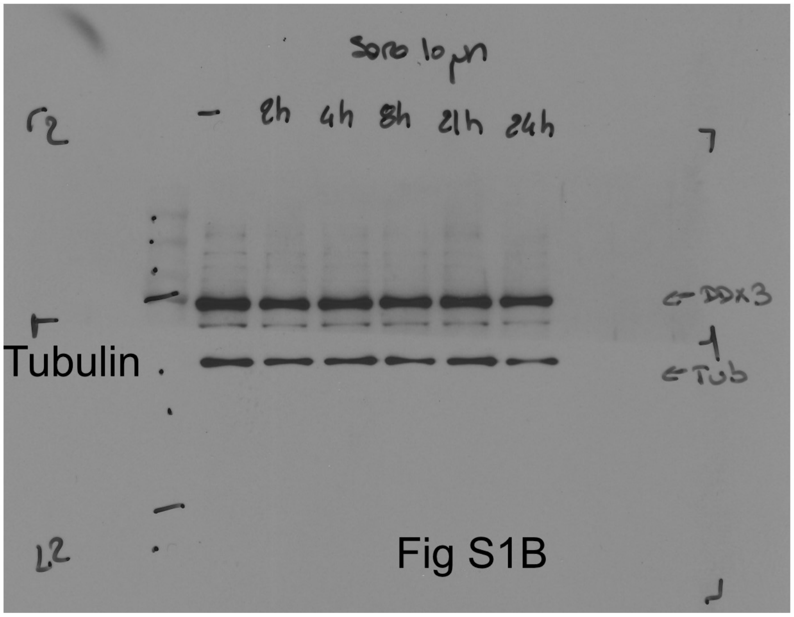

Fig S1B
